# Acknowledgement to Reviewers of *Nutrients* in 2019

**DOI:** 10.3390/nu12010273

**Published:** 2020-01-20

**Authors:** Nutrients Editorial Office

**Affiliations:** MDPI, St. Alban-Anlage 66, 4052 Basel, Switzerland

The editorial team greatly appreciates the reviewers who have dedicated their considerable time and expertise to the journal’s rigorous editorial process over the past 12 months, regardless of whether the papers are finally published or not. In 2019, a total of 3032 papers were published in the journal, with a median time to first decision of 16 days and a median time from submission to publication of 39 days. The editors would like to express their sincere gratitude to the following reviewers for their generous contribution in 2019:

A. Frame, LeighLin, Yen-ChungAalto, DanielLin, Yuh-FengAaron, GrantLindholm Bøgh, KatrineAaron, Jean E.Lindqvist, HelenAbate, GiuliaLindsay, AngusAbbasi-Maleki, SaeidLindsay, KarenAbbiss, HayleyLindshield, BrianAbbott, Catherine A.Liobikas, JuliusAbdel-Rahman, Manar E.Liou, Ying-mingAbdollahi, MehdiLipowska, MałgorzataAbdulkerim, ErogluLipson, Michael J.Abenavoli, LudovicoLisciani, SilviaAbeywardana, TharindumalaLiska, DeAnnAbondio, PaoloLittle, TanyaAboul-Enein, BasilLittlewood, Robyn A.Abraham, Bincy P.Liu, Chin-HungAbraham, Elizheeba ChristieLiu, ChunmingAbreu, SandraLiu, HaoyuAbutarboush, RaniaLiu, HexuanAbuzaid, AhmedLiu, JianAceves-Martins, MagalyLiu, JianghongAcosta, StefanLiu, JianmengActis, Giovanni CLiu, Jui-MingAdam, SumaiyaLiu, JunAdamczyk, PiotrLiu, JunxiuAdamowicz, JanLiu, KenAdams, Dawn WieseLiu, Lie-GangAdams, IngridLiu, LingAdams, James DavidLiu, Pei-YangAdamska-Patruno, EdytaLiu, ShichaoAddison, CliftonLiu, Shing-HwaAddo, O. YawLiu, YanAdekoya, Timothy O.Liu, YunfengAdeleye, BerniceLivesey, GeoffreyAdjé, FélixLlanos Chea, AlejandroAdriana, Estrada-BernalLlauradó, ElisabetAdriouch, SoliaLliberia, Mercè PallasAeddula, Narothama ReddyLLorente, FranciscoAgarwal, Mukesh M.Lo Coco, GianlucaAgarwal, PujaLo Giudice, AntoninoAgarwal, SumitLo Monte, Attilio IgnazioAgerbirk, NielsLo Muzio, LorenzoAghajafari, FaribaLo Scalzo, RobertoAglago, Elom KouassiviLo, Chunmin C.Agostoni, CarloŁobacz, AdrianaAgrawal, Sandeep K.Lobo, Glenn P.Agriopoulou, SofiaLochhead, Jeffrey J.Aguilar-Alvarez, DavidLockard, BrittanieAguilera-Tejero, EscolasticoLockridge, OksanaAgyei, DominicŁodyga-Chruścińska, ElżbietaAhmad, AamirLoewen, MicheleAhmad, FahimLogan, SreemathiAhmad, KhurshidLoh, KimAhmad, ShafqatLöhr, MatthiasAhmed, FaheemuddinLoiselle, Denis S.Ahmed, IshfaqLøken, Elin BAhmed, MavraLokesh, Belur RAhn, Mok-RyeonLokeshwar, BalakrishnaAhrens, Richard A.Loman, BrettAhuja, SumanLombardo, CaterinaAi, YongLombardo, MauroAinsworth, BarbaraLombó, FelipeAkers, JeremyLonardo, AmedeoAkter, Yeasmin AraLondon, EdraAlaimo, AlessandroLongo, RoccoAlam, Md BadrulLongo, ValterAlaofe, HalimatouŁoniewski, IgorAlbaladejo-Blázquez, NataliaLópez Lluch, GuillermoAlbanes, DemetriusLopez, David S.Albano, EmanueleLopez, NanetteAlcántara, Juan ManuelLopez-Bote, ClementeAldamiz-Echevarría, LuisLopez-Escamez, Jose A.Al-Dujaili, EmadLópez-Galán, BelindaAleksova, AnetaLópez-López, DanielAlexandre-Gouabau, Marie-cécile F.López-Pintor, ElsaAl-Fatimi, MohamedLord, JanetAlfieri, AndreinaLordan, RonanAlfieri, CarloLorenzo-Gómez, Maria-FernandaAli, Syed MustafaLostao, María PilarAlique, MatildeLosurdo, GiuseppeAl-Jawaldeh, AyoubLouis, PetraAllegaert, KarelLourida, IliannaAlloh, FolashadeLove, PennyAlmeida, Maria RosarioLovell, Amy L.Al-Mrabeh, AhmadLowden, ArneAl-Najim, WerdLowe, GordonAlsharairi, NaserLowe, NicolaAltemimi, AMMAR.BLu, Chi-HuaAltieri, BarbaraLu, Da-WenAltmae, SigneLu, JunAlvarez, Ana I.Lu, Kuo-ChengÁlvarez-Mercado, Ana IsabelLu, Louise WeiweiAlvarez-Sala-Walther, Luis A.Lucafò, MariannaAlvarez-Suarez, JoseLucarini, MassimoAlwahsh, SalamahLucas, Edralin A.Amarasena, NajithLucas, PatriciaAmati, FedericaLuchinat, ClaudioAmato, AntonellaLuden, Nicholas D.Amato, MarioLudy, Mary-JonAmbrogini, PatriziaLuévano-Contreras, ClaudiaAmbroszkiewicz, JadwigaLuis Ubago, JoseAmengual, JaumeLuk, Hui-YingAmigo-Benavent, MiryamLukaski, HenryAmodio, PieroLukaszuk, JudithAmorós, AsunciónŁukaszyk, EwelinaAmrein, KarinLuna, AurelioAn, Jeung HeeLund, JennyAn, WonsukLundberg, JonAnadón, ArturoLundman, BeritAnanieva, ElitsaLunn, WilliamAncìn-Azpilicueta, CarmenLuo, Xin M.Andersen, Barbara VadLuo, ZonghuaAndersen, Jens RikardtLupfer, ChristopherAndersen, Maria KarolineLupușoru, RalucaAndersen, VibekeLuscombe-Marsh, NatalieAndo, MasashiLustgarten, MichaelAndo, TakafumiLutjohann, DieterAndrade, IsabelLütjohann, DieterAndrade, JeanetteLutz, ThomasAndrade, Jeanette M.Lux-Battistelli, ChristineAndreeva, Valentina A.Lynch, HeidiAndres, AlineLynch, KrystalAndroutsos, OdysseasLynn, AnthonyAnestopoulos, IoannisLyons, GrahamAngela, HorvathLyons, JacquelineAngelino, DonatoM. Ney, DeniseAnifandis, GeorgeM’Koma, AmosyAnne-marie, CarreauMa, DavidAnnibale, BrunoMa, SihuiAnnunziata, AzzurraMa, YiyiAnnuzzi, GiovanniMa, Zheng FeeiAnsa, BenjaminMabhala, Mzwandile AndiAnsari, RaisMacchia, Paolo Emidio MidioAntcliffe, DavidMace, ThomasAntoine-Jonville, SophieMacedo, RodrigoAnton, StephenMachado, Mariana V.Anton, Stephen D.Machida, DaisukeAntonelli Incalzi, RaffaeleMaciejczyk, MateuszAntoni, RonaMaciejewska, DominikaAntosiewicz, JędrzejMaciejewski, GrzegorzAnyżewska, AnnaMack, IsabelleAoe, SeiichiroMacMillan, FreyaAparicio-Martinez, PilarMacRae, RodAppels, RudiMadar, A.Appleton, KatherineMadej, DawidArai, ToshiroMadzima, Takudzwa A.Araújo, João RicardoMaeda, HayatoAraujo, PedroMaehre, HanneAraujo, RicardoMaestro, MiguelArcher, DavidMagán-Fernández, AntonioArcher, EdwardMagiatis, ProkopiosArcher, NicholasMagierowski, MarcinArciero, PaulMagini, AlessandroArcot, JayashreeMagnone, MirkoAreal Borrego, FranciscoMagriplis, EmmanuelaArena, SimonaMahajan, SahilAreta, JoseMaher, JudeArguelles, SandroMahli, AbdoAridi, Yasmine S.Mahmud, IqbalAriyani, WindaMahmuda, Naila AlAriza, Maria TeresaMahoney, Sara E.Arjmandi, Bahram H.Mai, StefaniaArmah, Seth M.Mai, VolkerArmand, MartineMaia, CláudioArmitage, AndrewMaillard, VirginieArmstrong, HeatherMaione, FrancescoArnason, TerraMaity, SankarArnbjörnsson, EinarMajumdar, Adhip P. N.Aronsson, Carin AndrénMakabe, HidehumiArosio, PaoloMakarem, NourArthur, Susan T.Maki, KevinArtunc, FerruhMakowska, AgnieszkaArunkumar, AbhiramMakubuya, TimothyAsai, KazuhisaMalaguarnera, GiuliaAscoli Marchetti, AndreaMalaguarnera, MicheleAseervatham, JayaMalaguti, MarcoAshktorab, HassanMalatino, LorenzoAshley, PaulMalbert, Charles-HenriAsim, MuhammadMalby Schoos, Ann-MarieAsimakopoulos, ByronMalczewska-Lenczowska, JadwigaAslam, MohamadMalek, Angela M.Aspatwar, AshokMalfa, Giuseppe AntonioAssadi-Porter, Fariba M.Małgorzewicz, SylwiaAssaf-Balut, CarlaMalik, Rayaz A.Assari, ShervinMalin, AshleyAssi, SulafMalin, StevenAssink, MarkMalisova, OlgaAstbury, StuartMaloberti, AlessandroAstorino, ToddMaltais, MathieuAtaseven, BeyhanManai, FedericoAterini, StefanoManavifar, NegarAthyros, VasiliosMancini, AnnamariaAtkins, JaniceMancini, FrancescaAtkinson, JeffreyMandala, AshokAtkinson, SarahMandard, StéphaneAtkinson, StephanieMandava, NandaAttanasio, ChiaraMandò, ChiaraAtteritano, MarcoMandrioli, RobertoAttuquayefio, TukiMangels, ReedAubert, GregoryManiam, JayanthiAucoin, MoniqueManickam, NandiniAuguet, TeresaMann, Jake PAugustin, Livia SMannan, HaiderAuh, Joong-HyuckManoli, IriniAumeistere, LīvaManore, Melinda M.Aury-Landas, JulietteManthou, EiriniAustin, Erin EdwardManti, SaraAvallone, SylvieMantzioris, EvangelineAverill, MichelleMantzouridou, FaniAvery, JodieManville, RíanÁvila-Gandía, VicenteManzardo, AlessandroAvin, VijayManzo, CiroAvtanski, DimiterMaranesi, MargheritaAxelrod, Cara HannahMarangoni, FrancaAyalam, SrujanaMarasco, Lisa AnnAydemir, TolunayMarchesini, GiulioAyella, AllanMarcoli, ManuelaAyers, PhilMares, JulieAyonrinde, OyekoyaMaresca, MarcAyonrinde, Oyekoya T.Margalef, MariaAzarnia Tehran, DomenicoMargaritelis, NikosAzcárate-Peril, M. AndreaMargarucci, SabrinaAziz, Abdul RashidMarhuenda, JavierAzrad, MariaMaria José, Rodriguez LagunasAzushima, KengoMaría Rosaura, Leis TrabazoAzzi, AngeloMarian, ThomasAzzolino, DomenicoMarik, PaulAzzout-Marniche, DalilaMarinangeli, Christopher P.F.B Yeap, BuMarincola, Flaminia CesareBacaro, ValeriaMarini, Juan C.Bach Larsen, LotteMarino, JosephBachschmid, MarkusMarkaki, AnastasiaBackstrand, Jeffrey R.Markou, Kostas Β.Bacopoulou, FloraMarletta, LuisaBadenhorst, ClaireMarlicz, WojciechBadicu, GeorgianMármol, InesBae, JiyoungMarques, TatianaBaenas Navarro, NievesMarques-Lopes, IvaBahnson, EdwardMarquis, GraceBai, Li-YuanMarra, MaurizioBai, RenrenMarranzano, MarinaBailey, ReganMarriott, Bernadette P.Baird, Danielle LouiseMarrodán Serrano, María DoloresBaïz, NourMarrodan, DoloresBajaj, JasmohanMarroqui, LauraBajaj, PuneetMarshall, AutumnBajerska, JoannaMarshall, Sarah A.Bajka, BalazsMarsillach, JuditBajpai, VivekMarteau, PhilippeBaker, Brent A.Martelletti, PaoloBaker, JulienMartí, AmeliaBaker, SandraMartin Bermudo, Francisco ManuelBakht, MartinMartin, BrianBakillah, AhmedMartin, KeithBakker, Mieke H.Martin, LisaBalakrishnan, GayathriMartin, Stephanie L.Balantekin, KatherineMartin-Calvo, NereaBalcerczak, EwaMartindale, WayneBalcerczyk, AnetaMartinez Calejman, CamilaBaldassarre, Maria ElisabettaMartinez Sanz, Hose MiguelBallini, AndreaMartinez, HomeroBallmann, ChristopherMartinez, J. A.Bally, LiaMartínez-Cañamero, MagdalenaBaloni, PriyankaMartínez-Espinosa, Rosa MaríaBaltenweck, IsabelleMartínez-Galiano, Juan MiguelBälter, KatarinaMartinez-Guryn, KristinaBalusamy, Sri RenukadeviMartínez-Huélamo, MiriamBandyopadhyay, DhrubajyotiMartínez-Montemayor, Michelle M.Banerjee, Hirendra NathMartínez-Poveda, BeatrizBanerjee, JheelamMartínez-Ruiz, Nina Del RocíoBanerjee, KalpitaMartini, DanielaBanerjee, SudipMartín-Peláez, SandraBanfi, GiuseppeMartin-Rios, CarlosBang, Chang SeokMartins, Maria JoãoBaragetti, AndreaMartins, NataliaBarajas, MiguelMartucciello, GiuseppeBarak, YoramMarventano, StefanoBaratta, FrancescoMarverti, GaetanoBarbalace, Maria CristinaMarwarha, GurdeepBarbe, Anna GretaMarx, Joannes J. M.Barber, ElizabethMarzullo, PaoloBarber, TeresaMasaki, TakayukiBarbieri, ElenaMasala, CarlaBarbone, AlessandroMasarone, MarioBarbosa-Pereira, LetriciaMascherini, GabrieleBarbosa-Yañez, Renate LuzíaMascitti, MarcoBarca, AmilcareMasella, RobertaBárcena, CleaMaslin, KateBarchetta, IlariaMason, LauraBarchitta, MartinaMasot, OlgaBardají, EduardMassad, Salwa G.Bärebring, LinneaMassaro, MarikaBarichello, TatianaMassart, AlainBarker, AlanMasterson, Travis DBarlow, JonathanMastinu, AndreaBarnard, NealMastroianni, AntonioBarney, DavidMastroleo, FeliceBarnhill, KellyMasuda, JunkoBarone, MicheleMasuelli, LauraBarr, SusanMasulli, MariaBarrada, Juan RamónMatafome, PauloBarrajon, EnriqueMatarese, Laura E.Barratt, MichaelMatheson, BrittanyBarrea, LuigiMathews, Anne E.Barrera, Chloe M.Mathisen, Therese FostervoldBarrett, AnnMatias, CatarinaBarrett, Tessa J.Matias, Susana L.Barson, JessicaMati-Baouche, NarimaneBarszcz, MarcinMatinolli, Hanna-MariaBartelt, AlexanderMatkowski, AdamBarzilay, JoshuaMatoba, KeiichiroBasini, GiuseppinaMatsubara, KeiichiBasnet, SijanMatsui, TakaakiBasu, ArpitaMatsumoto, HiroyukiBaswan, SudhirMatsumoto, YoshinoriBatacan, RomeoMatsunaga, YutakaBattaglino, RicardoMatsushita, KensukeBattino, MaurizioMatsuzaki, EriBattram, DanielleMatsuzaki, KentaroBaum, JamieMatta, JoaneBaumann, MarcusMatteoli, GianlucaBazylko, AgnieszkaMatthys, ChristopheBazzano, Alessandra N.Mattii, LetiziaBeals, Joseph WMattioli, Anna VittoriaBean, ChristopherMattner, JochenBeard, MatthewMatwijczuk, ArkadiuszBeaudart, CharlotteMaugeri, AndreaBeaudreault, Amy R.Maugeri, Andrea GiuseppeBeberashvili, IliaMawhinney, ThomasBeccaria, FrancaMaximova, NataliaBecerra-Tomás, NereaMay, HTBeckett, EmmaMayengbam, ShyamchandBédard, AnnabelleMayeuf-Louchart, AliciaBegley, DavidMaynard, MariaBeharry, Kay D.Mayr, Hannah LBehringer, ErikMazidi, MohsenBei, RobertoMazur-Bialy, Agnieszka IrenaBeirowski, BogdanMazzilli, FernandoBejjanki, HariniMazzocchi, AlessandraBelenguer, AlvaroMcAdams, CarrieBelin De Chantemèle, Eric J.McArdle, HarryBellasi, AntonioMcBurney, MichaelBellini, GiuliaMcCabe, Laura R.Bellisle, FranceMcCarthy, ElaineBellissimo, NickMcCarthy, HelenBello, Nicholas T.McCarthy, Sinéad N.Bello-Espinosa, Luis E.McComb, JacalynBellumori, MariaMcCormack, LaceyBel-Serrat, SilviaMcCoy, PamelaBelstrøm, DanielMcCrickerd, KeriBelteki, GusztavMcDaniel, JohnBeltowski, JerzyMcGrath, Kathleen HBelval, LukeMcGregor, Neil R.Benatar, Jocelyne R.McGuire, HelenBendich, AdrianneMcGuire, ShelleyBenedetti, ElisabettaMchill, AndrewBenitez, SoniaMcHill, Andrew WBenito-Martin, AlbertoMcInnes, NataliaBenjamin-Neelon, Sara E.McJarrow, PaulBennett, Annemarie E.McKay, Naomi J.Benson, SarahMcKenna, Malachi J.Bentov, YaakovMcKenna, MaryBeppu, FumiakiMcKenzie, BriarBerding, KirstenMcKeown, NicolaBerendsen, AgnesMcLean, RachaelBerens, Pamela D.McMahon, GerardBerenz, AndrewMcManus, KirkBerg-Beckhoff, GabrieleMcMartin, KennethBerger, Gregor EmanuelMcMillin, MatthewBerghe, Carmina WandenMcNeil, DavidBergheim, InaMcNeil, JessicaBerhane, Hanna Y.McPherson, Nicole O.Bering, Stine BrandtMdegela, MselengeBernardazzi, ClaudioMearns, GaelBernardino, Raquel L.Medici, ValentinaBernhard, WolfgangMedina, F. XavierBernstein, MelissaMedina, GemaBerrett, Andrew N.Medina, Reinhold J.Berrington, JanetMedler, Kathryn F.Berry, Ann AndersonMeerza, Syed Imran AliBerryman, ClaireMeesters, DennisBerstain, JodiMeeusen, RomainBerteotti, ChiaraMehta, StutiBerthoud, Hans-RudolfMei, Hui-ChingBertini, MarcoMeier Zeman, DanielBertram, HanneMeinilä, JelenaBerube, Lauren ThomasMeisinger, ChristaBest, RussellMejía-Benítez, AuroraBetemariam, Senait AshenafiMekary, RaniaBeto, Judith A.Melaku, YohannesBettendorff, LucienMelaku, Yohannes AdamaBeyerlein, AndreasMele, LauraBezirtzoglou, EugeniaMelini, FrancescaBhagirath, DivyaMelini, ValentinaBharadwaj, ShishiraMellendick, KevanBharaj, PreetiMellody, K.T.Bhat, NehaMelo Van Lent, DeboraBhatia, JatinderMeltzer, Helle MargreteBhattacharjee, SonaliMenal, SusanaBhattacharya, AnannyaMéndez Mateo, InmaculadaBhattacharya, ApratimMendez, IanBhetwal, Bhupal P.Méndez, LucíaBhurosy, TrishneeMéndez-Álvarez, EstefaníaBiagi, CarlottaMeneguzzo, FrancescoBiagi, FedericoMeng, YuBiagini, GiuseppeMeng, Yuan XiangBiałek, AgnieszkaMenga, ValeriaBiałek, MałgorzataMenni, ChristinaBianchi, VittorioMenon, KavithaBibi, ShimaMensah, Enoch A.Bidault, GuillaumeMentella, Maria ChiaraBiddle, Gregory J. H.Mento, CarmelaBidwell, Amy J.Mercader, JosepBidzan, MariolaMercado, Carla IBielak-Żmijewska, AnnaMerchant, HamidBień, AgnieszkaMerendino, NicolòBigiani, AlbertinoMerighi, AdalbertoBigornia, Sherman JMerino Peláez, GraciaBijak, MichalMermier, Christine MBijak, MichałMerriman, TonyBikov, AndrasMerritt, Russell J.Bin, Yu SunMertens, EvelienBindels, JacquesMessias, AnaBiondi, BeatriceMessina, MarkBird, Julia K.Messini, Christina I.Birringer, MarcMessner, DonaldBiruete, AnnabelMessyasz, BeataBisbal, CatherineMethenitis, SpyridonBischoff-Grethe, AmandaMettler, SamuelBishayee, AnupamMetwally, MayadaBishehsari, FarazMetz, LoreBiskup, IzabelaMey, JacobBitok, EdwardMeyer, NannaBittner, Edward A.Meyerholz, David K.Bix, LauraMhd Jalil, Abbe MaleykiBizjak, DanielMian, CaterinaBizzaro, DeboraMicek, AgnieszkaBjørke Monsen, Anne-LiseMichaelidou, Alexandra-MariaBjørndal, BodilMichaëlsson, KarlBlaak, EllenMichalek, KatarzynaBlacher, EranMichels, AlexanderBlachier, FrancoisMichels, Alexander J.Blais, AnneMicillo, RaffaellaBlanchet, RosanneMicke, OliverBlanciardi, EmanuelaMiele, LucaBlanco-Gandía, M.CarmenMiele, MaraBlaney, SoniaMielenz, ManfredBlanton, CynthiaMielgo, JuanBlas, AlejandroMielgo-Ayuso, JuanBlasco-Lafarga, CristinaMierzyński, RadzisławBlasi, FrancescaMíguez, Jesús M.Blasiak, JanuszMikhailov, Theresa ABłaszczyk-Bębenek, EwaMiki, TakashiBlat, SophieMiki, YasuhiroBlazevic, IvicaMikkelsen, Bent EgbergBłażewicz, AnnaMikołajczak, BeataBleizgys, AndriusMilagro, FerminBlock, GladysMilan, AmberBlondin, StacyMilani, Gregorio P.Blouet, ClemenceMilani, Gregorio PaoloBlumenfeld Olivares, Javier AndresMilczarek-Andrzejewska, DominikaBo, SimonaMiletta, MariaBoard, MaryMillard-Stafford, MindyBoaz, MonaMillen, AmyBobeck, ElizabethMiller Jones, JulieBobowski, NualaMiller, JacquelineBobrowska-Korczak, BarbaraMiller, LaurieBocos, CarlosMillin, Paula M.Bodin, JohannaMills, JamesBodnar, ZsoltMills, KerryBogdanski, PawelMin, DuBogdański, PawełMin, Won LeeBohannon, Richard W.Min, Yang WonBohn, TorstenMinami, AkiraBohon, CaraMinami, YoichiBohrer, Benjamin M.Minato, Ken-ichiroBoland, MikeMinaya, DulceBold, JustineMine, YoshinoriBolignano, DavideMinihane, Anne MarieBolling, BradleyMinisola, SalvatoreBolton, KristyMinistrini, StefanoBommagani, ShobanbabuMinucci, SergioBonavina, LuigiMiotto, PaulaBoncinelli, FabioMiralles, BeatrizBond, Simon TMiranda, Andreia MachadoBondoc, IonelMiranda, JonatanBoneh, AvihuMirlohi, SusanBonilha, Heather SMisciagna, GiovanniBonilla, Diego A.Mishra, AbheepsaBonn, StephanieMisselwitz, BenjaminBonnländer, BerndMistura, LorenzaBonomini, FrancescaMitash, NilayBonuccelli, GloriaMitchell, LachlanBoquien, Clair-YvesMitchell, LanaBora, Puran S.Mithieux, GillesBorengasser, Sarah J.Mitre, NaimBorer, Katarina T.Mitrofanova, AllaBorghi, ClaudioMitsiakos, GeorgiosBoros, LászlóMiyagaki, TomomitsuBorradaile, NicaMiyata, JunBorradale, DavidMiyazaki, KoujiBorsani, ElisaMiyazaki, TeruoBortkiewicz, AlicjaMiyazaki, TetsuroBos, GerardMiyazato, HironariBosch, JaimeMiyoshi, TakekazuBose, TanimaMizuno, TooruBotet, FrancescMizuta, KentaroBotturi, AndreaMlak, RadosławBoucher, Barbera J.Mo, FeiBouillon, RogerMoat, StuartBourdel-Marchasson, IsabelleMobbili, GiovannaBourdon, EmmanuelModesti, Pietro A.Bourgeois, DenisMohamed, Azza H.Bourque, StephaneMohamed, IslamBoussios, StergiosMohamed, Salah A.Bovera, FulviaMohammed, AltafBovolin, PatriziaMohanty, Joy G.Bowen, DeborahMohd, Farooq ShaikhBowrey, David J.Moisa, SoniaBowron, AnnMoise, AlexanderBoy, ErickMojska, HannaBoyd, GaryMokadem, MohamadBoyer, Justin G.Molendijk, Marc L.Bozzetto, LutgardaMolina-Leyva, AlejandroBozzi, FabioMøller, GrithBraczkowski, RyszardMollo, ErnestoBradley, BethMoncayo, RoyBradshaw, PatrickMond, JonathanBraesco, VéroniqueMondal, PritishBragazzi, Nicola LuigiMonks, JeniferBrahma, SandrayeeMonsalve, MariaBrandhagen, MartinMontalcini, TizianaBrand-Miller, JennieMontaner, JoanBrandt, KirstenMonteagudo, AndreaBrann, Lynn S.Monteiro, CristinaBransfield, RobertMontejo, Juan CarlosBrantsaeter, Anne LiseMonteleone, IvanBrasó-Maristany, FaraMonteleone, PalmieroBraun, Frank K.Montesano, DomenicoBraverman, EricMontgomery, McKaleBravo Santos, RafaelMontoro, MiguelBrazeau, Anne SophieMontserrat-de La Paz, SergioBrech, DetriMoon, Il SooBrecht, MarcusMoon, Jin SooBrenna, J ThomasMoon, Jong-SeokBrennan, CharlesMoon, MinhoBreuner, Cora ColletteMoore, SallyBriana, Despina D.Moorehead, RogerBriançon, SergeMoores, CarlyBriand, LoicMoosmann, Dr. BerndBriassoulis, GeorgeMorabito, RosannaBrickley, BryceMorais, SamanthaBriend, AndréMorais, Tiago G.Briggs, Marc A.Morales Suárez-Varela, María M.Brigham, EmilyMorales, AlfonsoBril, FernandoMorales, RodrigoBrim, HassanMoranta Mesquida, DavidBrizuela, LeyreMorante, Juan José HernándezBroadhurst, C. LeighMorbidelli, LuciaBrombach, ChristineMoreau, RegisBronkowska, MonikaMoreira, Ana Sofia PereiraBrown, AndrewMorelli, LucaBrown, ClareMoreno Villares, JosèBrown, GeoffreyMoreno, J.J.Brown, KatieMoreno-Fernandez, JorgeBrown, Kerry AnnMoreno-Indias, IsabelBrown, LauraMoreno-Maldonado, ConcepciónBrown, LaVerne L.Moreno-Padilla, MariaBrown, RachelMoreno-Villares, Jose ManuelBrown, Robert J.Moretó, MiquelBrown, Ronald B.Moretti, DiegoBrown, VictoriaMorgante, GiuseppeBrownlee, IainMorgese, Maria GraziaBrueggen, Marie-CharlotteMori, KiyoshiBruneau Jr, MichaelMoriguchi, TakayaBrunner-Weinzierl, Monika ChristineMorin, MatiasBruns, HeikoMorino, KatsutaroBruserud, ØysteinMorita, ManabuBrustio, Paolo RiccardoMorita, NaokiBruun, SigneMoro, LoredanaBruyndonckx, LucMorris, CodyBryant, Camron D.Morris, NatashaBryniarski, KrzysztofMorriset, Anne-SophieBrytek-Matera, AnnaMorrissey, BridgetBrzeziński, MichałMorroni, FabianaBu, So YoungMorseth, Marianne SandsmarkBucca, CaterinaMosca, AndreaBucci, MarcoMosca, FabioBuchowski, MacMoser, OthmarBucki, RobertMoshage, HanBuckley, M.Mosoni, LaurentBuckley, SueMost, JasperBuczkowski, BartoszMota, João FelipeBuddington, RandalMotherway, Mary O’connellBudzyński, JacekMotoki, KosukeBuell, Jackie LMottawea, WalidBueno Dalto, DanyelMotyl, Katherine J.Bueno-Cavanillas, AuroraMounien, LourdesBuerkli, SimoneMounier, CatherineBührer, ChristophMoyle, LouiseBulla, JanMozas-Moreno, JuanBulla, RobertaMroz, ThomasBunn, DianeMu, QinghuiBuoite Stella, AlexMueller, Megan P.Burak, M.FurkanMuguerza, BegoñaBurdge, GrahamMuhle, PaulBurgess, JohnMujtaba, ShirazBurgess, John A.Mukherjee, AbirBurghardt, KyleMukherjee, RajibBurgos, Sergio A.Muldoon, Matthew FrancisBurke, DermotMuller, Manfred JamesBurke, LouisMuller, Patricia A. J.Burkhart, SarahMüller-Alcazar, AnettBurkhead, JasonMulloy, BarbaraBurnett, Alissa J.Munday, MichaelBurniston, Jatin GMunir, Kashif M.Burrows, RaquelMuniraj, NethajiBurton, Elvin ThomaseoMuniz, JuanBurton-Freeman, B.Munoz, ColleenBusetto, LucaMuñoz, MiguelBusnelli, MarcoMuñoz-Alférez, María JoséBustamante, María ÁngelesMuñoz-Torres, ManuelButel, Marie-JoséMuntané, GerardButler, Matthew P.Munusamy, ShankarButler-Dawson, JaimeMuraru, Iulia-DianaButt, Christopher M.Murff, Sharon H.Butts, ChrissieMurnen, Sarah K.Butts, CoryMuros Molina, José JoaquínCabantchik, IoavMurotomi, KazutoshiCabral, CeliaMurphy, MaureenCabrera Fuentes, Hector A. CabreraMurray, KathrynCadario, FrancescoMurray, MargaretCadenas, EnriqueMurtaugh, MaureenCahill, MichaelMuscat, DanielleCai, ChenxiMusch, MarkCai, DeminMusso, NataleCaiazzo, ElisabettaMustad, Vikkie A.Cairncross, CarolynMuthuramu, IlayarajaCairrão, ElisaMuzio, GiulianaCakebread, Julie A.Myasoedova, VeronikaCalabria, DonatoMyers, MarkCalamita, GiuseppeMyers, StephenCalani, LucaMyhre, Jannicke BorchCalapai, GioacchinoMyhrstad, Mari C.W.Calder, PhilipMyneni, AjayCalder, Philip C.Myszkowska-Ryciak, JoannaCaldo, Kristian MarkMzyaek, FawazCalkins, Kara L.Nagafuchi, YasuoCalle-Pascual, Alfonso L.Nagaraj, ViniCalle-Pascual, Alfonso LuisNagata, NaotoCalvo Lerma, JoaquimNagpal, RavinderCalvo Lobo, CésarNagwekar, JanhaviCamera, DonnyNagy, NadineCamilli, EmanuelaNahab, FadiCaminero, AlbertoNair, Gopika G.Camire, Mary EllenNair, SreenathCampbell, Grace J.Nair-Shalliker, VisaliniCampbell, SaraNakagawa, TakashiCampestre, CristinaNakajima, KatsuyukiCampo, Giuseppe MaurizioNakajima, KeiCanas, JoseNakamura, KozoCanavari, MaurizioNakamura, MiekoCancela, LeonorNakano, TakanariCandler, TobyNakao, ReikoCanene-Adams, KirstieNakata, SatoshiCangkrama, MichaelNakayama, KyosukeCano-Ibáñez, NaomiNalbandian, Harutiun M.Cansby, EmmelieNam, Ju-OckCantini, ClaudioNambiar, Dhanya K.Cao, ChuanhaiNanba, KazutakaCao, Jay JNandana, SrinivasCapaldo, B.Nandi, SaikatCaparros-Martin, JoséNanduri, RavikanthCapasso, RaffaeleNanri, HinakoCapel, FredericNaoki, AkazawaCapel, FrédéricNapierala, MartaCapitello, RobertaNarasimhulu, Chandrakala AlugantiCappellini, FrancescaNardelli, CarmelaCapuani, SilviaNarita, ShintaroCarabotti, MariliaNarkar, Vihang A.Caramaschi, DorettaNaruishi, KojiCarapella, LauraNasef, NohaCarbone, SalvatoreNaska, AndronikiCarbonell, TeresaNasser, JenniferCarbuhn, Aaron F.Natale, VincenzoCardenia, VladimiroNath, ArijitCardoso, Barbara R.Nath, ShubhankarCardoso, Susana M.Nathans, LauraCarella, Angelo MNatto, ZuhairCarey, ReneeNaughton, DeclanCargnin, SarahNaughton, ShaanCariello, MaricaNaumann, SusanneCarini, FrancescoNaumovski, NenadCarlberg, CarstenNavalta, JamesCarlomagno, ChiaraNavarrete-Munoz, Eva MariaCarlos, Ana RitaNavarro, Sandi L.Carlsen, Monica H.Navarro, VirginiaCarlsson, MartinNavarro-Alarcon, MiguelCarmine, Brian J.Navarro-Pérez, Carmen F.Carmona-Torres, Juan ManuelNavas-Carretero, SantiagoCarne, AlanNawirska-Olszańska, AgnieszkaCarner, Serafin CambrayNayanatara, Arun KumarCarnés, JerónimoNazmy, AydinCarobbio, StefaniaNeale, ElizabethCaron, RosemaryNeele, DellschaftCarotenuto, MarcoNeilson, AndrewCarpene, ChristianNejatinamini, SaraCarper, MichaelNelson, GreggCarpi, SaraNemzer, Louis RCarr, AmeliaNenna, AntonioCarr, AnitraNenna, RaffaellaCarriker, ColinNeola, MaggieCarrillo-Álvarez, ElenaNepal, Mahesh RajCarroll, ChadNeri, FlaviaCarson, BrianNescolarde, LexaCarson, Scott AlanNestares Pleguezuelo, Maria TeresaCarstairs, Sharon AnnNeto, António W. GomesCarta, GianfrancaNeubauer, KatarzynaCartmel, BrendaNevins, JulieCaruso, GianlucaNevzorova, Yulia A.Carvajal-Vidal, PaulinaNewby, RuthCarvelli, LuciaNexø, EbbaCasal, SusanaNeyraud, EricCasanovas, CarmenNg, DominicCasella, GiovanniNg, Hooi HooiCassarino, MaricaNg, Qin XiangCastada, HardyNguo, KayCastagna, MichelaNguyen, Long T.Castan-Laurell, IsabelleNi Lochlainn, MaryCastillo, CristianNibu, Ken-ichiCastorina, AlessandroNiccolai, AlbertoCastro, RitaNiccolai, ElenaCastro-Sanchez, ManuelNicholl, AnaliseCastro-Sepulveda, MauricioNichols, Buford L.Caswell, BessNicolai, MarisaCatalano, AntoninoNicolás, Francisco E.Caton, SamanthaNicoll, Amanda J.Cattaneo, AnnamariaNielsen, AndrewCatterson, James HNielsen, Mette SøndergaardCatto-Smith, AnthonyNielsen, Søren Drud-HeydaryCautela, DomenicoNieman, David C.Cavalcanti, ElisabettaNiemeier, AndreasCavaletti, GuidoNieri, PaolaCavallarin, LauraNiermann, ChristinaCedernaes, JonathanNiero, GiovanniCedó, LídiaNiess, Jan HendrikCena, HellasNikas, Spyros P.Ceolotto, GiulioNiknamian, SorushCeranowicz, PiotrNikolaidis, Pantelis T.Cermeno, MariaNikolakopoulou, Angeliki M.Cerniglia, LucaNikolaou, Charoula KonstantiaCervello, MelchiorreNikolic, MarinaCesareo, RobertoNiles, Richard MChaaban, HalaNilsen, Roy M.Chachay, Veronique S.Nilson, Eduardo A. F.Chacón Cuberos, RamónNilsson, AkeChae, Han-JungNilsson, StefanChakrabarti, SubhadeepNinomiya, KiyofumiChakraborty, MallinathNirala, NirajChakraborty, RajaNishi, MayumiChalari, EleannaNishi, Stephanie K.Chambers, EdwardNissinen, Markku J.Chamcheu, Jean ChristopherNistala, RaviChan, CatherineNlandu, Roger NgatuChan, DanielNobile, StefanoChan, JulieNobles, JamesChandon, PierreNoce, AnnalisaChandrashekhar, KshipraNofer, Jerzy-RochChang, Chen-KangNogueira, Rossana C.Chang, Chih-HsiangNolden, Alissa A.Chang, Chun-JuNolen-Doerr, EricChang, EugeneNomura, KyokoChang, Hsun-ShuoNonaka, KojiChang, Jer-MingNonino, CarlaChang, Jia-FengNonogaki, KatsunoriChang, Kee-LungNoonepalle, SatishChang, KoyinNordgren, Tara M.Chang, Sue-JoanNordhagen, StellaChang, susanNorman, KristinaChang, Yen-ChangNorris, KeithChao, Jane Chen-JuiNorsa, LorenzoChao, Wei-TingNorton, CatherineChaplin, AliceNorval, MaryChartoumpekis, DionysiosNotaras, Michael J.Chase, P. BryantNotarbartolo, MonicaChatelan, AngelineNotas, GeorgeChaudhri, VirendraNounagnon Frutueux, AgbanglaChauvea, PhilippeNounou, Mohamed IsmailChavatte, LaurentNouri, AriaCheemarla, NagarjunaNovío, SilviaCheemarla, Nagarjuna ReddyNovotny, JanetChelikani, PrasanthNowak, Felicia. V.Chelland Campbell, SaraNowicka, GrażynaChen, BinlingNowicki, GrzegorzChen, Chiao-NanNtila, SithandiweChen, Chung-Yen OliverNucci, DanieleChen, DanhongNugent, AnneChen, GuangpingNull, ClairChen, Guan-WenNüsken, EvaChen, GuoxunNuzziello, NicolettaChen, Jing-HsienNuzzo, DomenicoChen, JulianaO’Brien, Sarah H.Chen, Kong Y.O’Callaghan, Tom FChen, Kuan-FuO’Connor, Eibhlís M.Chen, Kuo-HuO’Connor, LaurenChen, Ling-WeiO’Gallagher, KevinChen, MinO’Halloran, Siobhan A.Chen, QiO’Leary, FionaChen, Rong-JaneO’Reilly, Kally C.Chen, SifanO’Sullivan, CathrynChen, TongO’Sullivan, MariaChen, Wei-YuO’Tierney-Ginn, Perrie FChen, XiaozhuoObana, AkiraChen, YanaObeid, RimaChen, Yi ChunObermüller, NicholasChen, Yi-MingObrenovich, Mark E. M.Chen, Yi-WenOchoa, JulioChen, Yu-FangOcker, MatthiasChen, Yung-ChihOdette, AimeeChen, ZhaoOdland, JonChen, ZhihangOdrowąż-Sypniewska, GrażynaChénais, BenoitOgasawara, ToruCheng, ChengOgra, YasumitsuCheng, ChunmingOgugua Asogwa, ClementCheng, Hong ShengOh, Soo YoungCheng, JiaOh, Yoon SinCheng, Kuan-ChenOhashi, NaroCheng, Meng-ChunOhlson, BodilCheng, XiOhnishi, KohtaCheng, Yuan-BinOhta, TakahisaChenoll, EmparOjetti, VeronicaCheriyath, VenugopalanOjima, KoichiCherkaoui Malki, MustaphaOkada, MuneyoshiCherniack, Evan PaulOkajima, HideakiChertok, Ilana R. AzulayOkochi, HitoshiCheung, Kei LongOkuda, MasayukiCheungpasitporn, WisitOkuda, NagakoChiang, Chien-HsiehOkumura, TadayoshiChiang, Meng-TsanOkunade, Adewole L.Chiang, Shu-HuaOláh, AttilaChiarioni, GiuseppeOldewage-Theron, WilnaChiavaroli, LauraOledzka, GabrielaChiba, TsuyoshiOlejar, Kenneth J.Chiefari, EusebioOlišarová, VěraChien, Li-YinOliva Pascual Vaca, Dr. ÁngelChien, Yi-wenOliveira, AndreiaChilds, CarolineOliveira, RuiChilibeck, PhilOliveros, ElenaChina, ArnabOliviero, TeresaChing, Tsui-TingOlivier-Van Stichelen, StéphanieChiou, Tzeon-JyeOlomu, IsokenChitchumroonchokchai, ChureepornOlova, NellyChiu, Wan-ChunOmagari, KatsuhisaChiva-Blanch, GemmaOmar, Syed HarisChmurzynska, AgataOmidian, KosarCho, ClareOng, Lin KooiCho, Jae YoulOng, QunxiangCho, SungeunOniga, IlioaraChoi, JungHunOniszczuk, AnnaChoi, Young WhanOno, HirakuChojnacki, JanOo, Win MinChong, MaryOracz, JoannaChoromanska, AnnaOrczyk-Pawiłowicz, MagdalenaChotta, Nikolas A.S.Ordonez Mena, José M.Chou, Lan-SzuOrenes Piñero, EstebanChouinard-Watkins, RaphaëlOrenes-Piñero, EstebanChouraqui, Jean-PierreOrf, IsabelChristakoudi, SofiaOrlich, MichaelChristensen, KristaOrtiz, J. BryceChristians, JulianOrywal, KarolinaChristides, TatianaOse, JenniferChristmann, ViolaOsella, Alberto RubénChristodoulou, Maria-IoannaOsganian, Stavroula K.Christoph, MaryOstachowska-Gasior, AgnieszkaChristophersen, ClausÖstenson, Claes-GöranChrousos, GeorgeOstojic, SergejChtourou, HamdiOstrowska, LucynaChu, Yueng-HsiangOtani, KeisukeChuang, Shih-YiOtsuka, IsaoChun, ReneOtsuka, YuzuruChun, SungkunOttaviani, JavierChung, EunheeOude Griep, Linda M.Chung, Hsin-FangOuguerram, KhadijaChung, Sang J.Ovesen, PerChung, SoonkyuOwen, AliceChung, Yong SukOyamada, MasahitoChurch, DavidOzorowski, GabrielChurchward-Venne, TylerÖzsürekci, CemileChutiyami, MuhammadPace, NelsonChycki, JakubPadhi, EmilyCiacci, CarolinaPadilla-Benavides, TeresitaCiappolino, ValentinaPádua, InêsCiardiello, Maria AntoniettaPagonopoulou, OlgaCiarimboli, GiulianoPaik, Hee YoungCibulskis, CatherinePaine, AnantaCiccone, MarcoPal Choudhuri, ShreoshiCiccone, Marco MatteoPalaniappan, BalasubramanianCicero, ArrigoPalanisamy, ArulselvanCicero, NicolaPali-Schöll, IsabellaCiciliot, StefanoPallavi, ManralCiebiera, MichalPallottini, ValentinaCieślińska, AnnaPalma, MariaCifelli, Christopher J.Palombi, LeonardoCilla, AntonioPalou, MarionaCioffi, FedericaPalsamy, PeriyasamyCioffi, IolandaPalumbo, RoccoCione, ErikaPalumbo, Vincenzo DavideCipolletti, ManuelaPamir, NathalieCirillo, MassimoPammi, MohanCirrincione, SimonaPammolli, AndreaCiszewski, Wojciech MichałPan, Min-HsiungClanchy, FelixPan, XiaoyueClark, BethPanagiotakos, DemonsthenesClarke, NeilPanagiotakos, DemosthenesClarke, Rachel E.Panahi, ShirinClassen, Hans-GeorgPanasiewicz, GrzegorzClayton, Zachary S.Panatala, RadhakrishnanClegg, MiriamPanek-Shirley, Leah MClemens, RogerPankratova, StanislavaClemente-Suárez, Vicente JavierPannaccione, AnnaClemente-Suarez, Vincente JavierPannala, AnanthClifton, PeterPantea Stoian, AncaCoan, P. M.Paolmino, OlgaCoates, PenelopePapadopoulou, AlexandraCocchi, MassimoPapakonstantinou, EmiliaCoccurello, RobertoPapanikolaou, YanniCock, CharlesPapathakis, Peggy CallaghanCoe, Christopher L.Papet, IsabelleCoelho, TracyPapi, AlessioCogswell, Mary E.Paquette, Marie-ClaudeCohen, RonaldParajuli, RajendraCohen, Tamara R.Paravisini, LaurianneColantuono, AntonioPardos Mainer, ElenaColberg, SheriPare, DramaneCole, MarshaParent, RomainCollado Yurrita, LuisParhofer, Klaus G.Collado, Pilar SánchezParis, HunterColleran, Heather L.Paris, JasonCollier, JasonPark, Clara YongjooCollins, JorjaPark, EunyoungColmán Martínez, MarielPark, Han-AColson, NataliePark, JeongjinColumbus, Daniel APark, Jin WooComan, Maria MagdalenaPark, Jun-BeomComelli, ElenaPark, Ki HwanComstock, SarahPark, Sang WonComulada, Warren ScottPark, SimonConde, JavierPark, Sook-HyunConde, SilviaPark, Sue K.Conkle, JoelPark, Yong-MoonConnaughton, Victoria P.Park, YukungConnolly, ChristopherParkar, Shanthi G.Conti, LuciaParker, Elizabeth A.Contreras, CristinaParker, Leslie A.Conway, JonathanParlesak, AlexandrCook, MatthewParodi, EmiliaCook, SarahParolini, CinziaCoombs, MelanieParoni, RitaCooper, JamieParr, EvelynCorazzari, MarcoParras-Rosa, ManuelCorbee, Ronald JanParrettini, SaraCorbi, GraziamariaParry, SionCordero, JoseParsanathan, RajeshCorella, DoloresParsons, LindaCorica, FrancescoPartridge, StephanieCormack, BarbaraPasanisi, PatriziaCornish, M. LynnPasca, SergiuCorpe, ChristopherPascual-Teresa, SoniaCorrado, AddolorataPashikanti, SrinathCorrea-Rodríguez, MariaPaslakis, GeorgiosCorsetti, GiovanniPaßlack, NadineCortés Castell, ErnestoPastor Vicedo, Juan CarlosCosola, CarmelaPastori, DanieleCosta, Ana RitaPaszkowski, TomaszCosta-Bauzá, AntòniaPataka, AthanasiaCostabile, GiuseppinaPatarrão, RitaCosta-Izurdiaga, AliciaPateiro, MirianCostante, GiuseppePatel, AnamikaCostantini, SusanPatel, ChloeCotelo, María Del Carmen SuárezPatel, DevenCottin, SarahPatel, Harnish P.Couch, RobinPatel, LiniaCoulson, SamanthaPatel, Yashomati M.Courel-Ibanez, JavierPathak, DhrubaCourel-Ibáñez, JavierPathirathna, Malshani L.Courtin, Christophe M.Patini, RomeoCoussons, PeterPatro-Małysza, JolantaCovasa, MihaiPatterson, KiraCox Sullivan, SheilaPatterson, Mindy ACox, Christopher R.Paulsen, PeterCoxson, PamelaPavlov, EvgenyCozar, IPavlova, NatalyaCraveiro, VandaPawliczak, RafalCrawford, Darrell H. G.Payne, AnneCrawford, SeanPazdro, RobertCrean, Angela J.Pearce, ElizabethCree-Green, MelaniePearson, James T.Cresbo Escobar, PaulaPedersen, Kim B.Cresci, Gail APeila, ChiaraCrespo, IrenePeiris, MadushaCrespo-Escobar, PaulaPekkala, SatuCrimarco, AnthonyPellegrino, DanielaCrispim, SandraPellegrino, RobertCrispin, PhilipPellicano, RinaldoCrocker, TheresaPellizzon, Michael A.Crofts, CatherinePeloso, Gina M.Crosby, KarenPeltomaa, ElinaCross, Carroll E.Pen, Joeri J.Crowley, JenniferPena, MariaCrowley, Shane V.Pena, Maria MarjoretteCrozier, SarahPendharkar, Sayali AjitCrus-Topete, DianaPeng, MeiCruz, RebecaPeng, MengfeiCsányi, GáborPeng, WenboCsiszar, AgnesPeng, Wen-HuangCsizmadi, IlonaPeng, YangCuadrado Soto, EstherPeng, Yi-JenCui, ChuanlongPenna, FabioCui, QingbinPenniston, KristinaCui, XiaoyingPentzold, StefanCukrowska, BoženaPenumarti, AnushaCumming, Mathew HoaniPepe, JessicaCunningham-Rundles, SusannaPepe, SalvatoreCurcio, GIUSEPPEPepeu, GiancarloCurtain, FelicityPepino, M. YaninaCusanelli, EmilioPercival, Susan S.Cutuli, DeboraPereira, Carlos DiasCzaja, KrzysztofPereira, Cidália DionísioCzaja-Bulsa, GrażynaPereira, Luciano J.Czarniecka-Skubina, EwaPereira, PatríciaCzerwonogrodzka-Senczyna, AnetaPereira, SóniaCzlapka-Matyasik, MagdalenaPereira-da-Silva, LuisCzyż, KatarzynaPereira-da-Silva, LuísD’adamo, StefaniaPerera, Liyanage P.D’Agostino, DonatoPérez-Badia, RosaD’Amato, MauroPérez-Burillo, SergioD’Anneo, AntonellaPerez-Cueto, ArmandoD’auria, EnzaPérez-Fuentes, María Del CarmenD’Elia, LanfrancoPérez-Gálvez, AntonioD’Elios, Mario MilcoPérez-Jiménez, JaraD’Lugos, AndrewPérez-Matute, PatriciaD’Occhio, MichaelPérez-Rodrigo, CarmenD’Souza, Randall FelixPerkins, AndrewDa Luz, Felipe Q.Perna, Dr. SimoneDa Paz, Vanessa Ribeiro FigliuoloPerna, SimoneDa Silva, A. MoreiraPero, RaffaelaDa̧browski, MariuszPeron, GregorioDacasto, MauroPerrin, Maryanne T.Dachineni, RakeshPerrot, TaraDachs, GabiPerry, RachelDaci, ArmondPerticone, MariaDaglia, MariaPeschel, Anne O.Dahdah, LamiaPestana, DiogoDahl, CeciliePeterfy, MiklosDahl, WendyPeters, John C.Dai, YuminPetersen, KristinaDai, ZhaoliPeterson, JuliaDai-Keller, JoyPeterson, LindaDaimiel, LidiaPetropoulos, Spyridon ADal Ben, MatteoPetruczynik, AnnaDal Monte, MassimoPetry, Clive J.Dalbeni, AndreaPetta, IoannaDallas, DavidPettersen, CarolineDalle Carbonare, LucaPettigrew, MarkDalton, BethanPeyer, KarissaDalton, CarolinePeyron, Marie-AgnèsDalton, SaynePezeshki, AdelDaly, AlisonPfeiffer, AndreasDaly, AnnePham, ThaoDamiani, GiovanniPham, Tho X.Damulevičienė, GytėPham, Thu HuongDandekar, AdityaPiacentini, Maria FrancescaDando, RobinPiasecki, JessicaDandona, PareshPiazzolla, GiuseppinaDanesi, FrancescaPiccoli, GiorginaDanezis, GeorgePiccoli, Giorgina BarbaraDang, WeiweiPiccolo, Brian D.Daniel, YvonnePieczyńska, JoannaDaniele, AntonellaPiemontese, LucaDanielewicz, AnnaPieretti, StefanoDanielson, Kirstie K.Pierre, Joseph F.Dankowiakowska, AgataPierzynski, JeanneDanner, Unna N.Pietrella, DonatellaDao, Maria CarlotaPifferi, FabienDarcy, JustinPigłowski, MarcinDarling, A.L.Pignitter, MarcDarmon, NicolePignotti, GiselleDarvesh, SultanPiknova, BarboraDarvin, MaximPilkington, Suzanne M.Das, AditiPilolli, RosaDas, UndurtiPilotto, AndreaDasarathy, SrinivasanPilz, StefanDasari, SurendraPinar, AnitaDatta, PoulamiPinel, AlexandreDave, SandeepPinent Armengol, MontserratDavis, JaimiePingitore, PieroDavison, KarenPinheiro, MarinaDavy, Kevin P.Pinho, Maria Gabriela M.Dawson, HarryPinkas, AdiDawson, JohnPinto, AlessandroDay, KaitlinPinto, AlexDe Baaij, JeroenPinto, Marina Marques Da Silva CabralDe Boer, AliePinto, PaulaDe Broe, Marc EPiotrowska, KatarzynaDe Bruyn, JuliaPirola, LucianoDe Chiara, FrancescoPirotta, StephanieDe Cosmi, ValentinaPistelli, FrancescoDe Filippis, FrancescaPitts, MatthewDe Filippo, GiampaoloPiva, TerrenceDe Geest, BartPivarunas, BernadetteDe Gennaro, LuigiPivovarova-Ramich, OlgaDe La Cruz, ErnestoPiwowarczyk, KatarzynaDe La Garza Puentes, AndreaPlano, DanielDe Lange, PieterPlastina, PierluigiDe Las Heras, JavierPlaza-Díaz, JulioDe Luna, AndreaPlessas, StavrosDe Magistris, LauraPlichta, MartaDe Marchi, ElisaPlochocki, JeffreyDe Marchi, MassimoPlogsted, SteveDe Martin, SaraPlotkin, Lilian I.De Masi, LuigiPlummer, VirginiaDe Paepe, BoelPochi, SubbarayanDe Pascual-Teresa, SoniaPochini, LorenaDe Pergola, GiovanniPocovi, GabrielaDe Salvo, MariaPoeggeler, Burkhard H.De Sanctis, Juan BPogorzelska-Nowicka, EwelinaDe Seymour, JamiePohl, SebastianDe Silva, FranklynPohlabeln, HermannDe Sire, AlessandroPolańczyk, AndrzejDe Smet, StefaanPolanska, KingaDe Souza Santos, RobertaPolesel, JerryDe Stefano, RosaPolge, CécileDe Steur, HansPoli, AndreaDe Vito, ElisabettaPomar, CatidoraDe Vries, JanPomeroy, JeremyDe Vries, JeannePoole, DavidDe, KuntalPopłoński, JarosławDean, ElizabethPorcelli, SimoneDe-Bandt, Jean-PascalPorebski, GrzegorzDeBiasse, Michele A.Porres, JesusDeBusk, RuthPortella, AndreDéchelotte, PierrePorter, Christine M.Dedoussis, George V.Posovszky, CarstenDeer, RachelPossidente, BernardDeibert, PeterPost, AdrianDeierlein, Andrea L.Pot, GerdaDekker, MarloesPothos, Emmanuel N.Del Giorno, RosariaPothuraju, RameshDel Pinto, RitaPotter, JuliaDel Rio, DanielePoudel, BarunDelafield-Butt, JonathanPoudel, Sher BahadurDelalle, IvanaPoulia, Kalliopi-AnnaDelarue, JacquesPourafshar, ShirinDelayre-Orthez, CarinePourcet, BenoitDelfine, SebastianoPowell, JonathanDelimont, NicolePower, Thomas G.Delisle, HélènePradeilles, RebeccaDella Torre, SaraPrado-Cabrero, AlfonsoDellaValle, Diane M.Praet, StephanDelort, LaetitiaPrakasam, SivaramanDelvin, Edgard E.Prats, María SoledadDe-Magistris, TizianaPratsinis, HarrisDemark-Wahnefried, WendyPravst, IgorDeme, PragneyPrescha, AnnaDemirdas, SerwetPrescott, MelissaDemmelmair, HansPrest, Melissa ADenizot, JéremyPrice, MennaDeo, PermalPrieto, IsabelDepalma, Ralph G.Prieto-Lloret, JesusDepeint, FlorePrince, BennieDepken, DianePrincipi, MariabeatriceDepmski, RobertPrinz, PhilipDepner, Christopher MichaelPrioleau, TemiloluwaDerbyshire, EmmaPritchard, Mary E.Derraik, José G.B.Priyadarshini, AnushreeDesai, AbhishekProbert, ChrisDesai, NiyatiProbst, YasmineDeshpande, RahulProcter, Sandra B.Desselberger, UlrichProietto, JosephDeth, Richard C.Prosser, Colin G.Deutsch, JonathanProtogerou, A.D.Devirgiliis, ChiaraProtudjer, JenniferDeWeese, RobinPrykhodko, OlenaDey, PrabuddhaPrzybyłowicz, KatarzynaDey, SoumyadeepPsomas, AkisDhariwala, MiqdadPtomey, Lauren T.Dharker, NachiketPu, ChristyDhillon, JaapnaPuga, Ana M.Dhindsa, SandeepPugh, JamieDi Bona, DaniloPujilestari, Cahya UtamieDi Carlo, MartaPulido, Olga MDi Donato, MarziaPulker, ClaireDi Giannantonio, MassimoPullen, NicholasDi Giuseppe, RominaPuma, Jini E.Di Mauro, MariaPumpa, KateDi Natale, ConcettaPunchard, Neville ADi Nunzio, MattiaPurdue-Smithe, AlexandraDi Pompo, GemmaPüschel, GerhardDi Primio, RobertoPutignani, LorenzaDi Renzo, LauraPutnam, William C. (Trey)Di Renzo, LiviaPuzianowska-Kuźnicka, MonikaDi Salle, AnnaPyzik, MichalDi Vita, GiuseppeQasem, WafaaDi Zazzo, ErikaQuadro, LoredanaDiamanti, AntonellaQuagliariello, VincenzoDiamond, DavidQuaglino, DanielaDias, Irundika H.K.Quante, MichaelDiaz, Eva C.Quattrocchi, AnnalisaDíaz, Juan JoséQuesada, IvanDíaz-Castro, JavierQuick, VirginiaDibaba, DanielQuigley, EamonnDickerson, RolandQuiles, JoanDieberg, GudrunQuintero-Florez, AngelicaDiehl, LindaQuinzii, Catarina MariaDieterich, WalburgaRaasmaja, AtsoDillon, Edgar LicharRaatz, Susan K.Dimas, KonstantinosRabionet, Silvia E.Dimidi, EiriniRabuazzo, Agata MariaDimitrellou, DimitraRaccuia, Salvatore AntoninoDing, LinRacette, SusanDing, YinyuanRachek, LyudmilaDioguardi, Francesco S.Rachek, Lyudmila I.Dioum, El Hadji M.Raczkowska, EwaDiretto, GianfrancoRadišauskas, RičardasDirks, Niek FRafiei, HosseinDjuric, ZoraRafiq, ShamailaDo Valle, Ítalo FariaRagavan, MukundanDobrowolski, HubertRaghunandan, MayaDobrowolski, PiotrRaheem, BamideleDobson, Adam J.Raheem, DeleDodd, MikeRahi, BernaDoddapattar, PrakashRahimi, FaridDoekes, GertRahman, SafikurDoering, TomRai, VikrantDoerr, CelesteRaina, KomalDolci, AlbertoRajakumar, KumaravelDoligalska, MariaRajaram, SujathaDomenicotti, CinziaRajesh Lenin, RajiDominguez, LigiaRalle, MartinaDomínguez, RaulRalli, MassimoDominguez, RubenRalston, Nicholas V. C.Domínguez-Perles, RaúlRamachandiran, IyappanDonato, RosarioRamadas, AmuthaDonato, Rosario FrancescoRamadori, GiulianoDonofry, ShannonRamalingam, NagendranDonohue, TerrenceRaman, MaitreyiDonovan, TimRamani, KomalDoo, MiaeRamani, SasirekhaDordevic, AimeeRamazzotti, MatteoDorling, JamesRamdas, Wishal D.Dörsam, Annica F.Ramdath, DanDos Santos, QueniaRamírez Sánchez, ManuelDotsikas, YannisRamírez, AngelDoyle, LornaRamirez, PatrickDoyle, ToddRamiro-Cortijo, DavidDragsted, LarsRamm-Pettersen, AnetteDrayna, DennisRamos, DomingoDreher, MarkRampanelli, ElenaDreher, Mark L.Randall-Simpson, JanisDresler, SławomirRangan, GopiDriver, AshleyRanieri, MariannaDrobnic, FranchekRanjan, KishuDroke, Elizabeth A.Rao, GouthamDrouin-Chartier, Jean PhilippeRao, RaghavendraDrozd-Dąbrowska, MarzenaRao, SheelaDrum, ScottRapi, StefanoDrzymała-Czyż, SławomiraRasmussen, HeatherDu, CaiganRastogi, DeepaDu, HuaidongRathi, NehaDu, YixingRaval, ManmeetDuarte, FátimaRavanidis, StylianosDuarte, T. L.Ravi, AnuradhaDube, JohnRawel, HarshadraiDube, PrabhatchandraRawla, PrashanthDubey, HarishchandraRayner, BenDuca, FrankRaz, OlgaDufour, Darna L.Razzaque, MohammedDugar, RohitRazzaque, Mohammed S.Dullaart, Robin P.f.Re, LambertoDullart, RobinRead, Scott A.Dumond, HeleneReale, MarcellaDuncan, AlisonRebeck, G. WilliamDuncanson, KerithReboul, CyrilDuntas, LeonidasRecchia, Daniela R.Duong, Tuyen VanRedan, BenjaminDupuis, KateReddivari, LavanyaDurazo-Arvizu, Ramon AReddy, ManjuDurazzo, AlessandraRedegeld, FrankDurazzo, MarilenaReed, KateDurkalec-Michalski, KrzysztofReed, SpenserDusso, AdrianaReglero, GuillermoDuszka, KalinaRego, RyanDutta, PranabanandaRegula, JulitaDuwaerts, CarolineReguła, JulitaDworatzek, PaulaRéhault-Godbert, SophieDyakin, Victor V.Rehman, MichaelDymarska, MonikaReicks, MarlaDzielska, AnnaReifsnider, ElizabethEarnest, ConradReiger, MatthiasEarnest, Conrad PReinders, IlseEastman, CreswellReiner, AgnesEbersole, Jeffrey L.Reinhardt, ChristophEck, Kaitlyn M.Reis, Amelia-PaulaEda, ShigetoshiReis, FlávioEdirisinghe, IndikaRej, AnupamEdiriweera, Meran KeshawaRelat, JoanaEdmonds, MichaelRemesar, XavierEduardo-Figueira, MariaRenner, WilfriedEdurne, Simón MagroRenwick, KerryEdwards, CatharinaResmark, GabyEdwards, Christine AnnRespino, MatteoEdwards, SarahRevel, Johana S.Eelderink, CobyReverter, EnricEgger, GarryRevuelta Iniesta, RaquelEggersdorfer, ManfredReynolds, AndrewEglseer, DorisReynolds, Clare M.Eicher-Miller, HeatherReznik, SandraEilertsen, Karl-ErikRhee, Ki-JongEkström, Eva-CharlotteRhoads, JonEkuni, DaisukeRhodes, JonathanEl Ghoch, MarwanRibaldone, DavideEl Hadi, HamzaRibaudo, GiovanniEl Midaoui, AdilRibeiro, ArturEl Shamieh, SaidRibeiro, DanielaElam, MarcusRibot, JoanEldeeb, MohamedRicci, AriannaEldeghaidy, SallyRiccio, PaoloEleftheriadis, TheodorosRiccioni, GrazianoElena, RicciRice, JohnEl-Esawi, MohamedRichard, CarolineElfvin, AndersRicordi, CamilloElgendy, MohamedRidberg, Ronit A.Eliaz, IsaacRiedel, BernhardEllervik, ChristinaRienecke, ReneeEllery, Stacey J.Rifler, Jean-PierreElli, LucaRigamonti, AntonelloElli, MarinaRigo, JacquesEllinger, SabineRiiser, Even SannesElliot, RuanRiley, MalcolmElliott, BradleyRimbach, GeraldElliott, CharleneRimms, EricElmarakby, AhmedRinaldo, NatasciaElmi, AlbertoRinninella, EmanueleEl-Seedi, HeshamRinsch, ChrisElsherbiny, MarwaRio, Carlos Martinez DelElshiewy, OssamaRios, FranciscoEl-Sohemy, AhmedRios-Covian, DavidElvira-Torales, Laura InésRishi, GautamElzen, Wendy P J DenRitchie, Lorrene D.Eme, PaulRiteau, NicolasEmerson, Dawn M.Ritz, ChristianEmerson, Sam RRiva, AlessandraEmmett, PaulineRivera, EleanorEngidawork, EphremRivera, TaniaEnglund Ögge, LindaRivero-Pérez, M. DoloresEnko, DietmarRizkalla, SalwaEnomoto, HirayukiRizo-Roca, DavidEnos, Reilly T.Rizzi, LauraErdman, John W.Rizzo, CaterinaEri, RajRizzo, GianlucaEricsson, MadeleneRoa, JuanEroğlu, AbdulkerimRobberecht, HarryErridge, ClettRoberts, JustinEscobar, JuanRoberts, KristenEscobedo Monge, Marlene FabiolaRoberts, MeganEscolà-Gil, Joan CarlesRobertson, KimberlyEsmaili, SaeedRobertson, KirstenEsposito, CiroRobinson, DonitaEsquerra-Zwiers, AnitaRobinson, EdwardEsse, RubenRobson, Shannon M.Estanis, NavarroRoca Llorens, MaríaEsteve-Romero, JosepRocca, CarmineEstevez, AnaRocha, Júlio CésarEstévez-Santiago, RocíoRocha-Ferreira, EridanEstevinho, Leticia M.Rocha-Rodrigues, SílviaEstruch, RamonRoche, EnriqueEsworthy, SteveRochfort, KeithEto, BrunoRockwell, MichelleEvans, E WhitneyRodda, Christine P.Evans, GethinRodgers, Kenneth J.Evans, RhobertRodrigo Saez, LuisEvin, GenevièveRodrigo, LuisEyles, DarrylRodrigues, FranciscaEzeamama, Amara E.Rodriguez Gómez, Juan MIguelEzekiel, UthayashankerRodriguez Sosa, Jose REzura, YoichiRodríguez Villanueva, JavierFabbricatore, MariantoniettaRodríguez, Alexander J.Fabiani, RobertoRodriguez, JoseFacchinetti, FabioRodríguez, Juan MiguelFahey, JedRodriguez, MarianoFairfield, CarolRodríguez-Almagro, Julián JavierFairweather, StephenRodríguez-García, CarmenFajardo, Val AndrewRodríguez-Gómez, IreneFam, JustineRodriguez-Mateos, AnaFan, QiongRodríguez-Mireles, SilviaFang, SungSoonRodriguez-Sanchez, NidiaFantuzzi, LauraRodríguez-Yoldi, María JesúsFarabegoli, FulviaRoe, AmyFarhat, GraceRoe, LianeFaria De Oliveira, MichelliRoe, MarkFaria, AnaRoetto, AntonellaFarinetti, AlbertoRog, JoannaFarmer, NicoleRogers, LisaFarran Codina, AndreuRogers, Lynette K.Farrell, Stephen W.Rogers, PeterFarzaneh, VahidRoggero, PaolaFava, CristianoRohde, CarmenFavero, GaiaRohira, AartiFayet, FlaviaRohn, SaschaFazio, AlessiaRohner, FabianFazio, Vito MicheleRohrmann, SabineFeas, XesusRojhani, ArezooFedosov, SergeyRojo-Martínez, GemmaFeillet-Coudray, ChristineRokavec, MatjazFeinman, RichardRolandsdotter, HelenaFelder, Robin A.Rolfo, AlessandroFeldmeyer, PierceRollin, EmmanuelFeresin, Rafaela G.Rollin, PatrickFerguson, DavidRomagnolo, Donato F.Feris, Edmond J.Romani, AndreaFerlazzo, NadiaRomecín, PaolaFernandes, Gustavo WerpelRomero-Saldaña, ManuelFernandes, RúbenRonchetti, SimonaFernandes, TitoRonsard, LaranceFernández A., LeónidesRonto, RimanteFernández Barbero, GerardoRoodenburg, AnnetFernández, IgnacioRoodenrys, StevenFernandez, Maria LuzRoot, MartinFernandez-Abascal, JesusRosa, Bruce A.Fernández-Bañares, FernandoRosa, DivellaFernandez-Jimenez, NoraRosado, CarlosFernández-Mateos, PilarRosário, RafaelaFernández-Ochoa, ÁlvaroRosato, EdoardoFernández-Sánchez, M.L.Roseland, JanetFernández-Villa, TaniaRoselli, MariannaFerramosca, AlessandraRosendahl-Riise, HanneFerrando, ArnyRoshanbinfar, KavehFerrar, JenniferRosinger, AsherFerrara, NicolaRosqvist, FredrikFerraro, Pietro ManuelRoss, MarkFerrell, Jessica M.Rossi, FilippoFerrer, Miguel D.Rossi, MauroFerrer-Gallego, RaúlRossi, Noreen F.Ferrie, SuzieRosso, NataliaFezeu, LeopoldRostami, KamranFiddler, Joanna LRostoff, PawełFielding, BarbaraRoth, Timothy C.Fields, DavidRothenberg, ElisabetFilannino, PasqualeRothschild, JeffreyFinelli, CarmineRouch, AlFineschi, VittorioRouche, ManonFinno, CarrieRouf, AnikaFinsterer, JosefRoumes, HélèneFiocco, DanielaRousseau, GuyFiorella, Kathryn JoanRousselot, BonnefontFioretti, BernardRoussis, Ioannis G.Fisher, GordonRowan, SheldonFitzgerald, John S.Rowland, Maisie K.Fitzgerald, KathrynRoy, Brian D.Flack, JohnRoy, KasturiFlack, KyleRozen, RimaFlaherman, Valerie JRóżyło, RenataFlak, Jonathan NRubio-Arraez, S.Flechtner-Mors, MarionRuddock, HelenFletcher, JaneRudzińska, MagdalenaFlidel-Rimon, OrnaRueda-Medina, BlancaFlores-Guerrero, Jose L.Ruiz, EmmaFlores-Quijano, Maria EugeniaRuiz-Litago, FátimaFlück, JoëlleRuiz-Ojeda, Francisco JavierFocsan, Alexandrina L.Ruiz-Santana, SergioFogacci, FedericaRumpunen, KimmoFogarty, AndrewRungratanawanich, WiramonFoligné, BenoitRunning, CordeliaFong, MackenzieRuoppolo, MargheritaFontaine-Bisson, BénédicteRupérez, Azahara IrisFonte, MariaRusciano, DarioFontés, MichelRush, Elaine C.Foong, Rachel E.Russell, FraserForbes, LauraRussell, SimonForbes, ScottRussi, SabinoForbey, Jennifer S.Russo, DanielaForhead, Alison J.Ruth, HarvieForman, MicheleRyabov, IgorFormenti, DamianoRyan, AnthonyForsse, Jeffrey S.Ryan, Elizabeth P.Forster, JamesonRybak, Leonard P.Forsum, Elisabet A.Rybicka, IgaFoster, Charles B.Ryffel, BernhardFourtounas, KonstantinosRytel, LilianaFoutch, BrianRytelewski, MateuszFox, MarkRzońca, EwaFrade, SaraRzymowska, JolantaFraga, Cesar G.Sabbatinelli, JacopoFragkos, KonstantinosSabeti, ElyasFram, MaryahSabia, MichaelFrame, Leigh A.Saccà, Sergio ClaudioFrancavilla, RuggieroSadak, Karim ThomasFrank, Craig L.Sadanaga, TsuneakiFrank, SamuelSadasivam, MohanrajFranklin, JanetSadowska-Krępa, EwaFranko, AndrasSaeedi, PouyaFranson, AndreaSaeki, KeigoFranzè, AnnamariaSaenz, MiguelFranzke, BernhardSáenz-Navajas, María-PilarFraser, DavidSaha, AchintoFrassetto, Lynda A.Saha, Amit KumarFrazer, David MSaha, SanjibFrediani, JenniferSahay, BikashFredolini, ClaudiaSahota, Tarsem S.Freire, MarceloSahrmann, PhilippFresán, UjuéSainsbury-Salis, AmandaFrezza, ClaudioSaint-Pol, JulienFriel, JamesSaisho, YoshifumiFriesen, CarolSaito, AkiFrigstad, Svein OskarSaito, TadaoFriso, SimonettaSaito, YukihiroFrugé, AndrewSakai, HiromichiFu, Pin-KueiSakai, KotomiFu, QiangSakai, TakahiroFueyo-Díaz, RicardoSakamuri, Siva Sankara Vara PrasadFujihara, HisakoSakasai-Sakai, AkikoFujimori, KoSakata, NaoakiFujioka, KazumichiSakhaee, KhashayarFujita, KojiSakharkar, PrashantFujita, YukiSakuma, KunihiroFujiwara, AyaSakurai, Toshihiro SakuraiFujiwara, TakeoSałaga, MaciejFukuda, MitsuruSalaheen, SerajusFukui, HirokazuSalahuddin, MelihaFukumoto, YoshihiroSalas-Huetos, AlbertFuller, ScottSalazar, GloriaFülöp, TiborSaleem, AyeshaFumagalli, LauraSalifoglou, AthanasiosFumeaux, Céline Julie FischerSalles, ChristianFunato, HiromasaSallinen, TaisaFüredi, AndrásSalman, MootazFurey, SinéadSalminen, Juha-PekkaFurman, LydiaSalomo, LouiseFürnsinn, ClemensSalonen, Bradley R.Furse, SamuelSaluk-Bijak, JoannaFusaro, MariaSalvatore, FrancescoFuu-Jen, TsaiSalvioni, CristinaG Gamage, DilaniSambri, VittorioGaby, JessicaSambuy, YulaGadd, HollySamczuk, PaulinaGaddam, Ravinder ReddySammarco, RosaGaesser, GlennSamoggia, AntonellaGaffney, KimSams, AnetteGaforio, JoséSanada, KiyoshiGahche, JaimeSanchez-Infantes, DavidGaitán, Adriana V.Sánchez-Muniz, Francisco J.Gajewska, DanutaSanchez-Niño, Maria DoloresGalanis, AlexisSanchez-Oliver, Antonio JGaleazzi, Gian MariaSanchez-Oliver, Antonio JesúsGaletti, ValeriaSánchez-Siles, Luis ManuelGalland, Barbara C.Sanders, DavidGallego, MartaSanders, ThomasGallo, MonicaSandhu, Vaneet KaurGálvez, JulioSandvik, PernillaGamazo, CarlosSangiao-Alvarellos, SusanaGammone, Maria AlessandraSankaran, Ganapathy SubramanianGanapathy, VadivelSankararaman, SenthilkumarGandini, SaraSantacroce, LuigiGandy, JoanSantamaria, RitaGanesan, PalanivelSantarpia, LidiaGanglmair-Wooliscroft, AlexandraSantofimia-Castaño, PatriciaGanguly, AvishekSantoro, NicolaGangur, VenugopalSantos López, Jorge ArturoGänzle, MichaelSantos, Débora Martins DosGao, ChunxuSantos-Gallego, CarlosGao, JieSantovito, DonatoGao, MingmingSantulli, GaetanoGao, XiangSanz, AlejandroGao, XuSanz-Paris, AlejandroGarbayo, InésSapone, AnnaGarbowska, MonikaSapudom, JiranuwatGarcés, CarmenSarabia-Cobo, Carmen MaríaGarcia Algar, OscarSarbini, ShahrulGarcia De Frutos, PabloSardu, CelestinoGarcia, AdaSarkar, AmritaGarcía, AngelaSarkar, DipayanGarcía, GabrielaSarker, MarjanaGarcía, MontserratSartini, MarinaGarcia-Alonso, JoséSartore, GiovanniGarcia-Bailo, BibianaSasaki, TsutomuGarcía-Conesa, María-TeresaSase, AjinkyaGarcia-de-la-Hera, ManoliSassi, YassineGarcía-Galbis, Manuel ReigSato, KeisakuGarcía-Martínez, EvaSato, KenjiGarcía-Martínez, InmaculadaSato, MasaoGarcia-Pardo, JavierSato, NorikoGarcía-Ródenas, ClaraSato, ShinGarcía-Sánchez, AsunciónSattler, Elisabeth Lilian PiaGarcía-Villalón, Angel LuisSaturnino, CarmelaGard, PaulSaul, DominikGardener, SamanthaSaunders, BryanGareb, BahezSautto, Giuseppe A.Gareri, PietroSavary-Auzeloux, IsabelleGarg, KawishSavastano, SilviaGarnacho-Castaño, Manuel VicenteSaw, Constance L.L.Garo, ElianeSawicka-Powierza, JolantaGarrido-Miguel, MiriamSayer, RichardGarrote-Adrados, José AntonioSayón, CarmenGartner, Lawrence MSazzini, MarcoGaston, SymielleScalco, AndreaGaudet, DanielScarano, AureliaGaul, DavidScavo, Maria PrincipiaGavrieli, AnnaScazzina, FrancescaGawron-Skarbek, AnnaSchaafsma, A.Gay-Quéheillard, JeromeSchaefer, MichaelGe, XiaodongSchag, KathrinGea, AlfredoSchalinskie, KevinGębski, JerzyScharnagl, HubertGeetha, ThangiahSchauss, AlexanderGęgotek, AgnieszkaScheer, Frank A.J.L.Geha, SamehScheers, Nathalie M.Geiker, Nina RWScheffler, ChristianeGeisler, CorinnaSchéle, ErikGélinas, PierreSchembre, SusanGemming, LukeSchenk, JeannetteGenco, Caroline AttardoSchenk, Jeannette M.Genoni, AngelaScherf, KatharinaGeraci, FabianaSchernthaner-Reiter, Marie HeleneGeraghty, AislingSchiano, ConcettaGerards, SanneSchiattarella, AntonioGerber, Leonard E.Schiavone, MarcoGerber, MarietteSchirhagl, RomanaGerkowicz, AgnieszkaSchirmer, BastianGeuns, Jan M.C.Schlader, ZacharyGevers, Dorus W.M.Schlichting, DeborahGhag, GauravSchmidt, LilianGharibyan, Anna L.Schmidt, MarkGhatak, ArindamSchmidt, MartinGhilli, MatteoSchoeler, Natasha E.Giacomello, EmilianaSchoenaker, Danielle AJMGiacomini, AndreaScholz-Kreisel, PeterGianfredi, VincenzaSchopfer, Francisco J.Gianni, Maria LorellaSchöpfer, JuttaGiannoni, PatriziaSchott, UlfGibbons, JeffSchöttker, BenGibert-Ramos, AlbertSchouteten, JoachimGibson, AliceSchulte, EricaGibson, Ann L.Schulze, JohannesGibson, LeighSchumacher, TracyGibson, PeterSchumacher, Tracy L.Gibson, SimoneSchüssler, SandraGiezenaar, CarolineSchutte, StaceyGifford, Janelle A.Schwartz, KennethGil-Cosano, Jose J.Schweitzer, DietrichGiles, CoreyScicchitano, PietroGill, HarsharnScilimati, AntonioGingras, VeroniqueScisco, JennaGiombini, LuciaScoditti, EgeriaGiordano, DanielaScott, DavidGiovannelli, PiaScott, Fraser W.Giovinazzo, GiovannaScott, HayleyGiraldo, PilarScott, Sam NGiralt, MartaScrafford, Carolyn G.Gisbert, Javier P.Scudiero, OlgaGitto, StefanoSeale, Lucia A.Giudetti, AnnaSears, BarryGiudici, Kelly VirecoulonSebastian, RhondaGiuffrè, Angelo MariaSebastiani, GiorgiaGiuliano, MichelaSechi, GianPietroGiuseppe, AnnuziataSeco, JesusGiustarini, DanielaSeconda, LouiseGiusti, Anna MariaŠeda, OndřejGłąbska, DominikaSedlacek, AbigailGlastras, SarahSedlak, LechGlendinning, JohnSeear, Kimberley H.Glynn, ErinSeetharaman, ShyamGnudi, SaverioSefton, JoEllenGoanvec, ChristelleSegatto, MarcoGodos, JustynaSegura-Garcia, CristinaGoel, Peeyush N.Sehrawat, ArchanaGoer, MaureenSeijas, RobertoGoglia, FernandoSeimon, RadhikaGogos, CharalambosSeino, ManabuGogulamudi, Venkateswara ReddySeino, YutakaGoh Boon Bee, GeorgeSekikawa, AkiraGolczak, MarcinSelland, CoreyGolombick, TerrySellayah, DyanGombart, AdrianSelya, ArielleGomes, Thayse NatachaSenesi, PamelaGomez De Agüero, MercedesSengenes, CoralieGómez Salgado, JuanSengupta, ArjunGomez, PierrickSengupta, BidishaGómez-Baya, DiegoSenoner, ThomasGómez-Guzmán, ManuelSeoane-Collazo, PatriciaGomez-Llorente, CarolinaSepulveda, Maria Alicia CarrilloGomez-Perez, Sandra L.Sergeant, SusanGomez-Pinilla, Pedro J.Sergi, ConsolatoGómez-Ruano, Miguel ÁngelSergi, DomenicoGómez-Vaquero, CarmenSerna, EvaGomollon, FernandoSerpa, JacintaGonçalves, CarlaSerra, FranciscaGonçalves, Elsa MargaridaSerra, GessicaGondek, AgataSerra, MonicaGong, XiaomingSerralheiro, Maria LuísaGonzalez Alvarez, IsabelSerrano, Jose C. E.González Jiménez, EmilioSestak, KarolGonzález Laredo, Rubén F.Seth, RataneshGonzález, JavierSethi, GautamGonzalez, Michael JSeto, Sai WangGonzález, SoniaShabnam, MominGonzalez-Casanova, InesShackelford, Rodney E.González-Minero, Francisco JoséShah, Rachana D.Gonzalez-Ochoa, GuadalupeShah, RuchiGonzález-Soltero, RocíoShah, ViralGonzález-Stuart, ArmandoShahar, DanitGoodla, LavanyaShahi, ShaileshGoodman, Richard E.Shaikh, Saame RazaGoodrich, JaclynShakerley, NicoleGordon, Eliza L.Shakibaei, MehdiGordon, MorrisShakya, AkhileshGordon, ScottShams-White, MarissaGorham, EdwardShang, QingsenGorman, ShelleyShangguan, SiyiGórnicka, MagdalenaShank, Lisa M.Gorrell, SashaShankar, EswarGorska-Ponikowska, MagdalenaShao, AndrewGosetti, FabioShapiro, Allison L. B.Gosselet, FabienSharma, BheshGoto, AtsushiSharma, Bhesh RajGoto, KatsumasaSharma, Girdhari M.Goua, MarieSharma, IshaGould, JacquelineSharma, KavitaGoulet, EricSharma, KumariGoya, LuisSharma, NaveenGraça, JoãoSharma, RohitGrace-Farfaglia, PatriciaSharp, David E.Graef, JenniferShashwat, SharadGraf, DanielaShearer, GregGrafenauer, SaraShechter, AriGramlich, LeahSheldrick, Michael P. R.Grammatiki, MariaShelley, MackGrammatikopoulou, Maria GShen, Wen-JunGranados-Principal, SergioShenoy, NirajGrant, GeorgeShepon, AlonGrant, WilliamSherman, PhilipGrases, FelixShertzer, Howard G.Gray, AndrewSherwin, Lee Anne B.Gray, ClintShete, VarshaGrech, AmandaSheth, SandeepGreen, AlannaShetty, AnandGreen, BenjaminShewale, SwapnilGreen, HilaryShi, QiangGreen, MarkShi, YuyanGreenhalgh, Andrew DavidShiao, S. Pamela K.Greenspan, PhillipShibata, HiroshiGreenwood, DarrenShibeeb, SaphaGreer, Frank RShidal, ChrisGreever, Cory J.Shidoji, YoshihiroGreeves OBE, JulieShigemura, YasutakaGregori, DarioShih, Chun-KuangGregory, StephenShim, Joon W.Gregory, Stephen L.Shimizu, MitsuruGrgic, JozoShimizu, TakahikoGrider, ArthurShimomura, YoshiharuGridneva, ZoyaShin, AesunGrieco, DomenicoShin, AndrewGrieger, Jessica A.Shin, Chong HyunGriffin, Nicholas W.Shin, Jung-AhGrigorescu, Elena-DanielaShin, SangahGrigorescu, FlorinShinde, TanviGrim, Clarence E.Shinichi, SekineGrimm, MarcusShinohara, MitsuruGrimm, Marcus OWShipman, KateGriñán-Ferré, ChristianShoben, Abigail B.Grochowski, CezaryShokal, UpasanaGrosicki, GregShokouh, PedramGrossi, GiancarloShook, AllanGrossini, ElenaShosha, EsraaGrosso, GiuseppeShrimpton, RogerGrubić Kezele, TanjaShriver, LenkaGruhlke, Martin C. H.Shukitt-Hale, BarbaraGruszka, Alicja MShukla, AlpanaGrygier, AnnaShvetsov, Yurii B.Grześkowiak, ŁukaszShypailo, Roman J.Gu, FengSiahanidou, TaniaGu, YianSicinska, EwaGuan, VivienneSiddiqi, Muhammad ZubairGuansheng, MaSiddiqui, MuhammadGuarino, MariaSieber, FritzGubatan, JohnSiega-Riz, Anna MariaGude, FranciscoSiegel, Jessica A.Gudey, Shyam KumarSiegel, Robert M.Gueimonde, MiguelSiegel, Sue RutherfordGueven, NuriSies, HelmutGuida, FrancescaSilk, LeslieGuinane, CaitrionaSilva Afonso, MilessaGuiné, RaquelSilva, Amelia MGunaje, JayaramaSilva, Ana FilipaGunasekar, Susheel KumarSilva, AndreGundamaraju, RohitSilvestre, Marta Filipa PaulinoGunia-Krzyżak, AgnieszkaSilvestri, CristoforoGunst, JanSilvestri, LauraGunton, JennySimmons, RebeccaGuo, Tai L.Simoes, SandraGuo, WeiminSimões-Wüst, Ana PaulaGuppy, FergusSimon, LaureGupta, AnshuSimon, Marie ChristineGupta, CharlotteSimona, Di FrancescoGupta, RajatSimper, TrevorGupta, SanjaySimpson, Melanie RaeGura, KathleenSimpson, SteveGuriec, NathalieSimu, Shakina YesminGustafson, Christopher R.Sinclair, AndrewGutiérrez-Juárez, RogerSindher, SayantaniGuzek, DominikaSingh, AnilGuzzardi, Maria AngelaSingh, AnupGuzzi, FrancescoSingh, ArashdeepGyawali, RabinSingh, ChandraH Shirvani, ArashSingh, HarsimranHa, AewhaSingh, M. V.Ha, Ki-TaeSingh, RajbirHa, Min-SeongSingh, Ravinder J.Haaparanta-Solin, MerjaSingh, RupeshHaas, VerenaSingh, SonalHaase, HajoSingh, VineetHabanova, MartaSingh, VishalHackett, DanielSingleton, ChelseaHaddad, EllaSioofy-Khojine, AmirbabakHafer, CarstenSipos, KatalinHagman, EmiliaSiracusa, RosalbaHahn, AndreasSiristatidis, CharalamposHailer, KatieSirohi, SunilHaining, Robert L.Sirover, William DHair, AmySirtori, CesareHair, Amy BoriskieSissener, Nini H.Haldar, SumantoSisti, GiovanniHall, SusanSivakumar, SushamaHall, Ulrika AnderssonSivanesan, IyyakkannuHall, WendySivaprakasam, SathishHalldorsson, Thorhallur IngiSkau Nielsen, TinaHallenius, FridaSkelton, Matthew R.Hallmann, EwelinaSkenderi, Katerina PHalloran, KieranSkoczylas, ŁukaszHalonen, Jaana I.Skopouli, FotiniHam, DanielSkórzyńska-Dziduszko, KatarzynaHam, Kyung-SikSkuratovskaia, DariaHamaguchi, MasahideSkypala, IsabelHamaguchi, ShogoSlavin, MargaretHamano, TadanoriŚliżewska, KatarzynaHambidge, K. MichaelSlominski, AndrzejHamlin, RobertSłomka, ArturHammes, Mary S.Slow, SandyHammond, Billy R.Ślusarczyk, SylwesterHampel, DanielaSlyvka, YuriyHamułka, JadwigaSmallfield, George B.Han, Der-ShengSmart, LauraHan, MingyuanSmeets, Paul A.M.Han, Seung HyeokSmetana, SergiyHan, Seung JinSmethers, AlissaHan, Sung NimŚmieszek, AgnieszkaHan, ZhiyongSmith, AndreaHanć, TomaszSmith, CarenHankir, Mohammed KSmith, ClaireHannibal, LucianaSmith, GordonHano, ChristopheSmith, Hazel AnnHansen, Mark BernerSmith, Jessica D.Hansen, MetteSmith, JohnEricHanson, JenniferSmith, Johneric W.Haque, InamulSmith, KennethHara, HiroshiSmith, Prof Stephen BHarasym, JoannaSmoluk-Sikorska, JoannaHarbige, Laurence S.Snelson, MatthewHardaway, AndrewSnipe, RhiannonHardman, Roy J.Snow, DeniseHardwick, James P.Snyder, RuthHardy, MelindaSobczyńska-Malefora, AgataHargreaves, IainSobeh, MansourHaring, BernhardSoe, KentHaring, RobinSogari, GiovanniHarmon, AlisonSoh, HosimHarnack, LisaSokal-Gutierrez, KarenHarnett, JoannaSokół-Łętowska, AnnaHaro, DiegoSokolowska, MilenaHaron, MonaSolanki, SumeetHarper, AlanSoler, CarlesHarray, AmeliaSolmi, FrancescaHarris, CarlaSoltysik, BartłomiejHarris, VanessaSomerville, VaughanHarris, William S.Somoza, BeatrizHarrison, FionaSong, In GyuHarrison, Jonathan S.Song, In-AeHart, Andrew RSong, JiajiaHart, Prue H.Song, MingHartanto, AndreeSong, PingHärtel, ChristophSong, SuHarthoorn, Lucien F.Song, YiqingHartmann, James X.Song, YoonJuHartmann-Johnsen, Olaf JohanSoni, DheerajHarton, AnnaSonja, LangHarville, EmilySopade, Peter A.Hasan, RaquibulSoriano, José MiguelHashemi, ShervinSornay-Rendu, ElisabethHashimoto, NaotoSospedra, IsabelHaskey, NatashaSotomayor, Camilo G.Haslam, DanielleSotos-Prieto, MercedesHassan, Amr AbdulfattahSotres-Alvarez, DanielaHassan, Amro B.Soueidan, AssemHassan, IbrahimSoulika, AthenaHatziantoniou, SophiaSoundharrajan, IlavenilHausken, TrygveSowndhararajan, KandhasamyHautbergue, ThaisSozańska, BarbaraHavas, KristinaSpadafranca, AngelaHawinkels, LukasSpagnuolo, RoccoHay, IanSpanakis, Elias K.Hay, WilliamSpanier, BrittaHayashi, Mariko KatoSpano, GiuseppeHaycraft, EmmaSpartalis, EleftheriosHayes, KCSpeck, MelanieHaynes, AshleighSpeed, JoshuaHeaney, SusanSperanza, GiovannaHeath, AliciaSpiegler, JulianeHeberden, ChristineSpiller, RobinHebert, JamesSpisni, EnzoHebert, James R.Spiteri-Cornish, LaraHeer, MartinaSpitznagel, Mary BethHegazi, RefaatSpoerl, DavidHegazi, Refaat A.Sponsiello, NicolaHeianza, YorikoSpradley, Frank T.Heil, Sandra GSprague, MatthewHeilbronn, LeonieSpriano, GiuseppeHeilmayr, DietlindeSpyridon N., KarrasHeinen, MirjamŠrámek, JanHeinrich, KatieSrinivasan, ShantiHeiss, CindySrinivasan, VijayHelajärvi, HarriSriwastva, MukeshHelena Siegel, MarciaStaats, StefanieHeller, GerwinStacchiotti, AlessandraHeller, JanoschStacewicz-Sapuntzakis, MariaHellinger, RolandStachowska, EwaHellmann, Jason L.Stadlbauer-Köllner, VanessaHelm, SusanStadler, DianeHelms, EricStadnik, JoannaHelmy, Yosra A.Staege, Martin S.Hemilae, HarriStahl, Chad H.Hemmingway, AndreaStaiano, AnnamariaHendrich, SuzanneStallings, VirginiaHendriksma, HarmenStamm, RoseHenes, Sarah T.Stanford, Fatima CodyHenjum, SigrunStanford, KristinHenkel, JaninStanford, StephanieHenneman, PeterStanforth, Philip R.Hennessy, ÁineStange, KatjaHerbert, AnthonyStanhope, JessicaHerda, AshleyStaniek, HalinaHerforth, AnnaStanirowski, Paweł JanHermansen, KjeldStanislawski, Maggie A.Hermes, Gerben D AStanley, Jone A.Hernández, AdrianaStannard, StephenHernández, ÁngelStarck, CarleneHernandez-Alonso, PabloStarościak, WojciechHernandez-Hernandez, OswaldoStarowicz, MałgorzataHernández-Socorro, Carmen RosaStauber, Rudolf E.Herpertz-Dahlmann, BeateStauffer, Melissa E.Herránz, HéctorStaunstrup, Nicklas HeineHerrera, EmilioStavropoulos-Kalinoglou, AntoniosHerrick, KirstenStawny, MaciejHerrmann, MarkusStefan, NorbertHerrmann, WolfgangStefanaki, CharikleiaHervás, GotzoneStefanidou, Maria E.Hess, JulieStefler, DenesHetland, GeirStehle, PeterHew-Butler, TamaraStein, Jesse A.Hewelt-Belka, WeronikaSteinhauser, JohannHewlings, SJSteinhoff, U.Heyn, PatriciaSteinmeyer, JuergenHibi, MasanobuStelmach-Mardas, MartaHietavala, Enni-MariaStenbeck, GudrunHigaki, KazutakaStengel, AndreasHigashi, YukihitoŠtěpánek, LadislavHigashimura, YasukiStephanie J, WeinsteinHiggins Neyland, MaryStepien, Karolina M.Higgins, Mary L. MeckStettler, ChristophHii, Charles S.Stevens, RebekahHill, AileenStewart, MariaHill, Alison M.Stiuso, PaolaHill, CameronStixova, LenkaHiller, Amie L.Stobdan, TseringHillier-Brown, Frances C.Stocks, JoanneHimeno, SeiichiroStoecker, BarbaraHimmerich, HubertusStojek, MonikaHimoto, TakashiStokes, Caroline S.Himotoe, TakashiSt-Onge, Marie-PierreHines, Erin PiasStorcksdieck Genannt Bonsmann, StefanHingle, MelanieStornaiuolo, MarianoHingorani, RittuStote, KimHintze, KorryStout, JeffreyHiraoka, SakikoStrandvik, BirgittaHirasaka, KatsuyaStrasser, BarbaraHirasawa, AkiraStrati, FrancescoHirko, KellyStrelnikov, KuzmaHissa, BarbaraStroebele-Benschop, NanetteHivert, Marie-FranceStuetz, WolfgangHM Temme, ElisabethStulnig M, ThomasHo, VincentStuper-Szablewska, KingaHoane, MichaelStur, ElaineHoban, RebeccaSturm, Brigitte N.Hobbs, MatthewStylianou, MichalisHobin, ErinSu, YuanjieHocher, BertholdSuárez-Llanos, José PabloHodge, A. M.Subbaraj, Arvind K.Hoeller, UlrichSubramani Paranthaman, BalasubramaniHoerr, RobertSubramanian, VeedamaliHofer, Matthias D.Sudfeld, ChristopherHoffman, NolanSugawara, AkihiroHoffman, RichardSugimoto, NaotoshiHoffmann, PeterSuh, HyunGyuHogarth, LeeSuh, Jun GyoHoge, AxelleSukseree, SupawadeeHögger, PetraSukumar, DeepthaHohensinner, PhilippSukumaran, Sunil KumarHojo, HironoriSuleria, HafizHokama, TomikoSuleria, Hafiz Ansar RasulHolefors, AnnaSuliburska, JoannaHolland, Angelia MaleahSuliburska, Joanna MariaHolland, JustinSuliga, EdytaHollenbach, MarcusSumczynski, DanielaHolley, ClareSun, ChengwenHollis, BruceSun, Feng-HuaHolscher, HannahSun, MingKuanHolt, DarcySun, QianHolt, RobertaSun, XiaofeiHolvoet, PaulSun, YangboHommel, Jonathan D.Sunami, YoshiakiHong, JaeheeSundaram, KruthikaHong, Jiann-RueySundaram, UmaHong, Mee YoungSundaramoorthy, PasupathiHooda, JagmohanSundekilde, Ulrik K.Hooper, LeeSundin, Olof H.Hopkins, LauraSung, Jia-YingHopperton, KathrynSuominen, SakariHoque, Mohammed ZiaulSurguchov, AndreiHoracek, TanyaSuriano, FrancescoHorne, Benjamin D.Suryadevara, VidyaniHorowitz, MichaelSutter, CarolynHorrigan, Louise A.Suwalska, AleksandraHorstmann, AnnetteSuzuki, KatsuhikoHorswill, Craig ASuzuki, TakuyaHorwath, CarolineSuzuki-Kerr, HarunaHoshino, DaisukeSuzutani, TatsuoHosie, AnnmarieSvoboda, MartinHosking, SarahSwain, JamesHosokawa, YoshitakaSwamy, GaneshHosomi, RyotaSwan, EmilyHossain, Md EkhtearSwoap, StevenHossain, SharminSylow, LykkeHosui, AtsushiSymonds, Michael E.Hou, RuixueSyurina, ElenaHou, Wen-ChiSzczepanek, KingaHouston, Mark C.Szczerbinski, LukaszHoward Wilsher, StephanieSzewczuk, Myron R.Howarth, GordonSzewczyk, Nathaniel J.Howe, Caitlin GSzewczyk-Golec, KarolinaHoy, Mary KatherineSzilagyi, AndrewHruz, Paul W.Szkudelska, KatarzynaHsieh, Kuen-ChangSzlagatys-Sidorkiewicz, AgnieszkaHsieh, Tusty-JuanSzostak-Węgierek, DorotaHsieh, Vivian Chia-RongSzychlinska, Marta AnnaHsieh, Yi-ChenSzymandera-Buszka, KrystynaHsu, Sheng-DerSzymanowska, UrszulaHsu, Shu-haoTabara, YasuharuHu, AndyTaber, ChristopherHu, GuoliTacconelli, StefaniaHu, JianTada, HayatoHu, JianzhongTaddese, HenockHu, QiwenTaft, Diana HHu, TianTaft, TiffanyHu, YingTagliaferri, SaraHu, YuanTaglietti, ValentinaHuang, Chi-ChangTAGUCHI, KENSEIHuang, Chih-YangTaharoom, Saman KhalesiHuang, Hui-ChunTajiri, YujiHuang, Kuang-TzuTakahama, UmeoHuang, Li-TungTakahashi, HideyukiHuang, Men-ChungTakahashi, MasakiHuang, Paul Li-HaoTakahashi, YumikoHuang, Shih-YiTakaki, AkinobuHuang, Wen-ChungTakata, TomoakiHuang, XiaohuTakata, YumieHuang, Yi-ChiaTakaya, JunjiHuërou-Luron, Isabelle LeTakayuki, TsukuiHughes, PatrickTakenaka, YukinoriHughes, SamanthaTakeuchi, ToshihideHuh, Jin WonTako, EladHuling, JenniferTalamonti, EmanuelaHull, HollyTalati, ZenobiaHulthén, LenaTalbot, ShawnHummel, SandraTaleb, NadineHuneau, Jean FrançoisTallapaka, Shailendra B.Hung, Kuo-ChinTambe, MitaliHung, Szu-ChunTamura, YukinoriHunsberger, MonicaTan, Bee KangHur, Yang-ImTan, IsabellaHurrell, RichardTan, XiaoHurtado, Ghaffar AliTana, ClaudioHurwitz, LaurenTanabe, KatsuyukiHuston, Robert K.Tanaka, NaoroHwang, Daniel Liang-DarTanaka, SatoshiHwang, In KooTanaka, ShigehoHwang, Lee-ChingTanaka, TakujiHyder, Salman M.Tanasova, MarinaHydery, TasminaTanda, Maria LauraHyldig, GretheTang, JonathanHylemon, Phillip B.Tang, MinghuaHynes, KristenTang, YaoIacobazzi, VitoTangney, Christy C.Iacone, RobertoTaniguchi, KoheiIacovou, MarinaTanner, S.BoboIaniro, GianlucaTannous, KathyIbars, MariaTanui, Collins K.Ibrahim, FandiTanumihardjo, SherryIbrahim, KindaTao, Meng-HuaIchikawa, HiroshiTappia, ParamjitIchimura, HiroshiTaraboletti, AlexandraIde, KazukiTarantino, GiovanniIentile, RiccardoTareen, SamarIgarashi, KazueiTarleton, Emily K.Igarashi, KiharuTarragon Cros, ErnestoIglesia, IrisTartey, SarangIglesias-Osma, Maria CarmenTasevska, NatashaIizuka, KatsumiTauber, JamesIkeda, KaoriTavori, HagaiIkemori, AtsukoTawfik, AmanyIkura, YoshihiroTawia, SusanIlag, Leodevico L.Tayie, FrancisIlesanmi-Oyelere, Bolaji LilianTaylor, Carla G.Illangakoon, U.ErankaTaylor, Christopher A.Imai, ToshioTaylor, RachaelImamura, FumiakiTaylor, Rachael M.Imm, Jee-YoungTeigen, LeviInafuku, MasashiTeixeira, Ana MariaInfante, MarcoTeixeira, CátiaInglada-Pérez, LucíaTelang, SuchetaInglis, Julia E.Telle-Hansen, Vibeke H.Inman, SharonTempfer, HerbertInns, StephenTemple, NormanInohara, NaohiroTen Haaf, DominiqueIoakeimidis, IoannisTeng, AndreaIonita, PetreTenore, GianIppolito, Joseph E.Teodoro, JoãoIqbal, Tariq H.Teotia, PoojaIreton-Jones, CarolTepper, Beverly J.Irie, JunichiroTerpou, AntoniaIsaacs, AnnaTersey, Sarah A.Isaza Martínez, José HipólitoTeschke, RolfIseppi, LucaTessem, JefferyIshibashi, KenichiTessema, MasreshaIshihara, KengoTesta, AlexanderIshii, HidekiTestai, LaraIshimi, YoshikoTeusch, NicoleIshimoto, TakahiroTeves, María EugeniaIslam, Md. TabibulThackray, Alice EmilyIsmail, RitaThakali, KeshariIsola, GaetanoThanikachalam, KannanIspoglou, TheocharisTheiler-Schwetz, VerenaIto, FumiakiTheorell-Haglöw, JennyIto, NaokiTheradiyil Sukumaran, AnurajIuliano, RodolfoTherese Fostervold, MathisenIuliano, SandraThil Smith, Adam AlexanderIvana, VucenikThoene, MelissaIves, StephenThoene, MichaelIwahori, ToshiyukiThoma, ChristianIwai, AtsushiThomas Craig, Kelly J.Iwanicki, AdamThomas, ChristoforosIwasaki, YusakuThomas, D. TravisIyer, ChitraThomas, JencyIzawa, Kazuhiro P.Thomas, JenniferIzawa, TakeshiThomas, TiffanyIzquierdo Pulido, MariaThomas, WayneIzycki, DariuszThompson, Campbell H.Izydorczyk, BernardettaThompson, MichaelJ. Barba, FranciscoThompson, SandraJ. Steffen, KristineThomsen, Jesper S.Jaakkola, JohannaThomson, David M.Jaaro-Peled, HannaThomson, JessicaJabłońska-Trypuć, AgataThomson, Neil C.Jack, RhonaThornley-Brown, DenyseJackson, KristinaThreapleton, Diane ErinJacob, NoamThurl, StephanJacobs, DavidThuzar, MoeJadeja, RavirajsinhThyagarajan, BaskaranJaeger, RalfTian, YuanJaehne, Anja KathrinTiao, Mao-MengJafari, JafarTicha, AlenaJagannathan, RamTicinesi, AndreaJäger, PiaTie, Jian-KeJaggupilli, AppalarajuTierney, AudreyJahan-Mihan, AlirezaTiidus, PeterJahidul, IslamTimchenko, NikolaiJahn, SteffenTimmerman, KyleJahns, LisaTinsley, GrantJain, AnshikaTiwari, VijayJaite, CharlotteTkaczewska, JoannaJajtner, AdamTobias, Deirdre K.Jakobek, LidijaTobin, DerekJakobsen, MogensTobina, TakuroJakobusic Brala, CvijetaTocmo, RestitutoJalava, KatriToledo Atucha, EstefaníaJalil, Abbe Maleyki MhdToledo-Corral, ClaudiaJalili, ThunderTomassoni, DanieleJalili-Moghaddam, ShabnamTomaszewska, EwaJalo, ElliTomayko, EmilyJamar, GiovanaTomczykowa, MonikaJameson, EleanorTomkin, GeraldJanda, ElzbietaTomkin, Gerald HJandacek, Ronald J.Tomlinson, David J.Jang, Byoung KukTomofuji, TakaakiJang, SunginTong, JessicaJanga, Srikanth ReddyTongaonkar, PrasadJanikowska, GrażynaToniolo, LuanaJansen, Erica C.Tonkin, EmmaJanssens, SharonTonolo, GiancarloJape, GayatriTonorezos, EmilyJara Palacios, María JoséToomer, OndullaJarman, MeganToorie, AnikaJarrett, CatherineTopa, GabrielaJärvenpää, Eila P.Toribio-Mateas, MiguelJasdanwala, SarfarazTörn, CarinaJawad, Muhammad A.Torquati, LucianaJawinski, PhilippeTorregrossa, Ann-MarieJaworowska, AgnieszkaTorres Fuentes, CristinaJayaraman, AnushaTorres, NimbeJeandet, PhilippeTorres, SusanJeanes, YvonneTørris, ChristineJeckel, Kimberly MTosh, SusanJeffery, ElizabethTotosy De Zepetnek, JuliaJelalian, ElissaTouil-Boukoffa, ChafiaJelinic, MariaTovar, JuscelinoJelski, WojciechTovit, RosenzweigJelsma, JudithTovoli, FrancescoJena, Prasant KumarTownsend, StevenJeney, ViktóriaTraina, GiovannaJenkins, DavidTraka, MariaJenner, KatharineTran, Anh NhatJensen, Jørgen DejgårdTran, KimJensen, Megan E.Tran, PhuJeon, Byeong HwaTrautwein, ElkeJeppesen, Per BendixTravers, Jeffrey BryantJerzak, Malgorzata MariaTreccani, GiuliaJeżewska-Zychowicz, MarzenaTremblay, AngeloJi, Bao-anTrenell, MichaelJi, PengTrentini, AlessandroJi, YuelongTriantafillidis, John KJiang, BaishanTriantafyllou, KonstantinosJiang, WeiTrif, MonicaJiang, XinyinTrijsburg, LauraJiao, LiTrinchese, GiovannaJilani, HannahTrindade, RicardoJiménez - Ortega, VanesaTripathi, ManishJimoh, FlorenceTripepi, GiovanniJimoh, Oluseyi F.Tripicchio, GinaJin, MirimTripolt, Norbert JoachimJitnarin, NattineeTriunfo, StefaniaJob, KathleenTroisi, JacopoJoe, MillwardTrommelen, JornJohansson, IngegerdTroncone, RiccardoJohnson, AlexanderTronel, ClaireJohnson, Colin P.Trovato, FrancescaJohnson, IanTrovato, GuglielmoJohnson, Ian T.Trovato, Guglielmo M.Johnson, Jay E.Trovik, JoneJohnson, Kelly E.Troy, LisaJohnson, Lance A.Truman, EmilyJohnson, RichardTrumpff, CarolineJohnson, Sarah A.Tryggestad, JeanieJohnston, CarolTsafantakis, NikolaosJohnston, Kelly L.Tsai, Chia-WenJohnstone, Alexandra M.Tsai, Ming-HorngJohnstone, DanielTsai, Ying-ChiehJones, MarjorieTschon, MatildeJoo, Nam-SeokTsekoura, MariaJorda, Marta AlegretTseng, Ching-JiunnJørgensen, Anders PalmstrømTseng, Yu-HuaJosef, KapfhammerTsermpini, Evangelia EiriniJoseph, JacobTsivgoulis, GeorgiosJoseph, Joshua JTsofliou, FotiniJoseph, SusanTsopmo, ApollinaireJoshi, KamalTsukamoto, HayatoJospe, MichelleTsuruta, TakeshiJoubert, MichaelTucci, SaraJoven, JorgeTuck, CarolineJówko, EwaTucker, RobinJoyce, Susan A.Tucker, WesleyJozwiak, MathieuTudor, KateJu, Man KiTugault-Lafleur, ClaireJu, Sang-YhunTugault-Lafleur, Claire N.Juan, WenyenTurck, DominiqueJukić Peladić, NikolinaTurner, JustineJukic, Anne Marie Z.Turner, Raymond ScottJulve, JosepTurpin-Nolan, SarahJumbo-Lucioni, PatriciaTurrini, AidaJung, Hyun ChulTurroni, SilviaJung, Ji HoonTuvdendorj, DemidmaaJurewitsch, BrianTveden-Nyborg, PernilleJůzl, MiroslavTye-Din, JasonJyväkorpi, Satu KTyrka, MirosławKaaja, RistoTyszka-Czochara, MalgorzataKaar, Jill LandsbaughTzakos, AndreasKaarniranta, KaiTzvetkova, PavletaKabil, HadiseUberti, FrancescaKabisch, StefanUdagawa, JunKachroo, PriyadarshiniUddin, Golam MezbahKaczocha, MartinUebanso, TakashiKaczyńska, KatarzynaUeda, NorishiKadioglu, OnatUehara, MarikoKadowaki, TakashiUfnal, MarcinKaeffer, BertrandUlatowski, LynnKagawa, MasaharuUllrich, MatthiasKageyama, HakutoUllrich, ReinerKagiya, TadayoshiUlrich, CraigKahane, RémiUmar, ShahidKaido, ToshimiUmmarino, SimoneKaikkonen, JariUnger, EricaKajarabille, NaroaUnno, KeikoKaklamanou, DaphneUnoki-Kubota, HiroyukiKale, AbhijitUpadhyay, Kiran K.Kales, StefanosUpadhyaya, JasbirKalhan, SatishUrashima, TadasuKalin, Marcia F.Urbanelli, LorenaKaliora, Andriana C.Uribarri, JaimeKallas, ZeinUrra, José MiguelKalliopi, KotsaUrrechaga, EloisaKaluza, JoannaUrrestarazu, ElenaKam, LynnUsai Satta, PaoloKamath, SandipUsui, KenjiKamenidou, IreneUusitalo, UllaKamiloglu, SenemVaarno, JenniKaminski, RafalVacante, MarcoKaminskyy, VitaliyVacanti, NathanielKamolz, Lars-PeterVacca, MicheleKampmann, UllaVaccaro, OlgaKamprath, SagarVafeiadi, MarinaKanamori, TakaoVafiadaki, ElizabethKanauchi, MasaoVaidya, AmitaKanda, AtsushiVaisman, BorisKanda, NaokoVaitkus, AntanasKandala, Ngianga-BakwinVaittinen, MaijaKandar, RomanValdivielso, Jose ManuelKandikattu, HemanthValencak, TeresaKandylis, PanagiotisValentová, KateřinaKang, ChenValenzuela, Pedro LKang, Hee Y.Valenzuela, RodrigoKang, InhaeValera-Gran, DesiréeKang, Jae SeungValerio, GiulianaKang, KyungsuValitutti, FrancescoKang, Tae-HongValles, Soraya L.Kang, Tong HoValtchev, PeterKang, WonkuVamanu, EmanuelKang, Young-HeeVan Amburgh, MichaelKanno, YoshihikoVan Baak, MarleenKanno, YosukeVan Ballegooijen, Adriana JKantake, MasatoVan Ballegooijen, HanneKantartzis, KonstantinosVan Beusecum, Justin P.Kantono, KevinVan Bodegraven, Ad AKapoor, Mahendra P.Van Buiten, CharleneKapravelou, GaryfalliaVan Buul, Vincent J.Kapsokefalou, MaríaVan De Heijning, Bert J. M.Kapur, NeerajVan De Walle, DavyKarachaliou, MariannaVan Den Heuvel, EmmyKarakochuk, CrystalVan Der Bend, DaphneKarastergiou, KalypsoVan Der Gaag, EllenKarayannakidis, PanagiotisVan Der Heijden, Gert-JanKarayiannakis, AnastasiosVan Der Merwe, MarieKarcz, DariuszVan Der Putten, Gert-JanKardassis, DimitrisVan Der Zee, EddyKarhunen, LeilaVan Dronkelaar, CarlieneKarl, J. PhillipVan Dyke, KnoxKarlsen, MicaelaVan Egmond, LieveKarlsson, ErikVan Gorkom, Gwendolyn N. Y.Karlsson, HakanVan Herck, Mikhaïl A.Kårlund, AnnaVan Hoek, BartKarner, CourtneyVan Leeuwen, Sander S.Karolkiewicz, JoannaVan Poppel, MireilleKasarda, DonaldVan Putten, MaaikeKashfi, Khosrowvan Schothorst, Evert M.Kass, Lindsy S.Van Strien, TatjanaKastaun, SabrinaVan Vliet, StephanKatakura, YoshinoriVan Zanten, Arhtur R.H.Kato, HiroyukiVandenplas, YvanKatsagoni, Christina N.Vander Wal, JillonKattner, LarsVanderhoof, Jon A.Katzmarzyk, PeterVanderpas, JeanKaufman, Beth D.VanDusseldorp, TrishaKaufman-Shriqui, VeredVanella, LucaKauppinen, Tiina M.Vanmierlo, TimKaur, GunveenVapaatalo, HeikkiKaur, JapneetVarela, Lourdes MKaurani, LalitVarela-López, AlfonsoKavanagh, Katherine F.Varela-Silva, Maria InêsKavanaugh, DevonVargas, Jorge HKavecan, IvanaVargas-Murga, LilianaKaviani, MojtabaVarinen, AleksiKavle, JustineVasilevsky, NicoleKawai, ShinichiVassanelli, AuroraKawai, Vivian K.Vatner, Daniel FKawakami, YukiVattem, DhirajKawakita, DaisukeVaughn, Mathew.A.Kawamukai, MakotoVaz, JosianaKawamura, AkiraVaziri, NosratolaKawamura, TakujiVecchione, CarmineKawamura, TomoyukiVelasco, InésKawanami, DaijiVelazquez, RamonKawasaki, KiyonoriVeltkamp, ChristianKayser, BengtVemula, HarikaKazemi, MaryamVemuri, RavichandraKean, James DVenditto, VincentKeane, DavidVenegas, EvaKebede, Mihiretu M.Veneri, Diana A.Kędzierska, EwaVenkatesan, ThiagarajanKeith, Jill F.Venkatesh, SiddarthKeller, HeatherVenn, BernardKeller, KathleenVenner, EricKelly, Owen J.Venskutonis, Petras RimantasKelly, Terri-AnnVenter, CarinaKendig, MichaelVentura Ribeiro, RosileneKenichi, SakuraiVepsäläinen, HennaKennedy, GerardVerbeke, KristinKennel, JulieVerd, SergioKenney, Erica L.Verdijk, LexKennon-McGill, StefanieVerdú, ElenaKephart, WesleyVergadi, EleniKephart, Wesley C.Vergara, DanieleKerksick, ChadVerma, AnilKerr, JessicaVerma, NirmalKershaw, JonathanVermiglio, FrancescoKerver, JeanVerneau, FabioKeshteli, Ammar HassanzadehVerpeut, JessicaKesztyüs, DorotheaVerri, TizianoKevany, KathleenVeskoukis, Aristidis S.Key, Chia-ChiVest, Katherine E.Khachik, FredVetere, AmedeoKhaddaj-Mallat, RayanVetrani, ClaudiaKhadka, ManojVézina-Im, Lydi-AnneKhallouki, FaridVia, Michael A.Khambadkone, Seva GVidot, HelenKhan, KrishnenduVierucci, FrancescoKhan, NaghmaViggiano, DavideKhan, Zafar K.Vigna, LuisellaKharat, SuhasVilarrasa, NuriaKhare, SharadVilela, SofiaKharrazian, DatisVilla, ChiaraKhatri, KshitijVillalba, Josè ManuelKheradpezhouh, EhsanVillalonga, PriamKhosla, AashimaVillani, AnthonyKhoury, DaliaVillarini, AnnaKido, JunVilstrup, HendrikKiełczykowska, MałgorzataVincentini, OlimpiaKieliszek, MarekVinet, AgnesKiesswetter, EvaVinoy, SophieKietzmann, ThomasVinson, JoeKiewiet, GeaVirag, Jitka A.I.Kikuchi, HidehikoVirella, DanielKilari, SreenivasuluVirginio Agostoni, CarloKilian, CarolinVirsolvy, AnneKim, Dong SeonVirtanen, JyrkiKim, Hee-SunVirtanen, Suvi M.Kim, Hye-KyeongVisentin, GiulioKim, HyesookVisioli, FrancescoKim, Jae KyeomVissers, MargreetKim, JaehanViswanathan, PreetiKim, JaeheeVita, RobertoKim, Jin-KyungVitale, Jacopo A.Kim, Jong-SangVitale, MarilenaKim, Jung-HaVitale, Salvatore GiovanniKim, Kil-SooVitiello, PeterKim, KirangVitoria Miñana, IsidroKim, Kyoung SooVitturi, DarioKim, Mee KumVivas, SantiagoKim, Myeong OkVlassopoulos, AntonisKim, Ok-SuVliet, Stephan VanKim, SoJungVo, GiauKim, SunheeVogt, Leonie M.Kim, Tae ImVolicer, LadislavKim, WookyungVollmer, LoriKim, YanghaVollmer, RachelKim, YeonsooVolpato, MileneKim, YongsooVon Hurst, P.Kim, YoonaVon Schacky, ClemensKim, Young-SangVon Wright, AtteKim, YuriVoortman, TrudyKimball, Samantha M.Vormann, JürgenKimber-Trojnar, ŻanetaVorontsova, IreneKimura, Ken-ichiVoziyan, PaulKinsky, Suzanne MVoznesenskaya, AnnaKipp, MarkusVutukuri, Naga MalleswariKirby, AnnieWaalen, JillKirk, BenWada, YasuakiKishikawa, NaoyaWaddell, JaylynKishimoto, YoshimiWadolowska, LidiaKiss, AnnaWägner, Ana M.Kiss, RobertWagner, CarolKitatani, KazuyukiWagner, JeremyKlaassen, TimWaitt, CatrionaKlarskov, KlausWaldherr, KarinKleber, Marcus E.Waldhör, ThomasKlein, Gordon L.Walker, MarjorieKlein, LauraWalker, Meegan A.Klepsatel, PeterWallace, AlisonKlettner, AlexaWallert, MariaKlims-Zacas, DorothyWalters, JamesKling, Pamela J.Walters, MatthewKling, Samantha M.R.Walton, KathrynKlinger, MarianWalyaro, DJKlonizakis, MarkosWalzem, RosemaryKnaapila, AnttiWang, BoKnez, MarijaWang, ChaoChenKnight, KathyWang, Cheng-hsuKnight-Agarwal, CathyWang, DanlingKnipping, KarenWang, DongqingKnott, RachelWang, Fan-FenKo, Hae-JinWang, GaofengKo, Jiunn- LiangWang, Grace Y.Ko, Shun-YaoWang, GuorongKobayashi, Eri H.Wang, Jie (Jane)Koch, AlexanderWang, KaiKoch, Timothy R.Wang, KimberleyKoch, WojciechWang, Ming-FuKodali, MaheedharWang, OuKoehler, KarstenWang, PeizhongKoenig, LauraWang, PengchengKoh, Ho-JinWang, QunKohler, Lindsay N.Wang, TongKoirala, NiranjanWang, XianfengKok, WouterWang, XiangdongKoka, Sai SudhaWang, YifeiKokoszyński, DariuszWang, Yi-FenKolarik, Andrew J.Wang, YunguanKoletzko, BertholdWang, ZenengKolota, AleksandraWang, ZhenpingKolsteren, PatrickWang, ZhijunKolwicz Jr., Stephen C.Wanigatunga, Amal AsiriKomai, MichioWankhade, Umesh D.Komeili, AminWanrooij, PaulinaKomici, KlaraWard, AimeeKomnenov, DraganaWard, LeighKondadi, Arun KumarWard, Marie-LouiseKones, RichardWard, NicolaKonishi, HirotakaWarden, Stuart J.Konrads, ChristianWardenaar, FlorisKonstantinidou, ValentiniWark, PetraKopczynski, MichalWarner, Brad W.Koppe, Janna G.Warner, John O.Koppe, LaetitiaWarrington, Junie PKorczak, RenataWarzecha, ZygmuntKorhonen, Marko T.Washburn, SteveKorivi, MallikharjunWatanabe, MikikoKorkalo, LiisaWatkins, Adam J.Kornprat, PeterWatkins, DavidKorre, MariaWatson, PoppyKoshinaka, KeiichiWatson, Wendy L.Kosik-Bogacka, DanutaWawer, AnnaKosinsky, Robyn LauraWawrzyniak, AgataKostecka, MalgorzataWax, BKosters, AstridWeaver, ConnieKostomitsopoulos, Nikolaos G.Weaver, MatthewKotani, KazuhikoWeber, JanetKoturbash, IgorWedi, EdrisKounis, Nicholas GWeems, MaryKoutelidakis, Antonios E.Weerasekara, Permani C.Koutelou, EvangeliaWei, GanKoutoukidis, DimitriosWei, YonglongKovanecz, IstvanWeiland, TraceyKovatcheva-Datchary, PetiaWeiler, HopeKowal, MałgorzataWeiss, GunterKowalska, MalgorzataWeiss, ScottKowalski, RadosławWeiss, SiegfriedKoyama, YuWeiss, Thomas S.Kozlowski, Michael R.Wendell, Stacy GelhausKrähenbühl, StephanWendin, KarinKrajewska-Włodarczyk, MagdalenaWendy, WismerKrajka-Kuźniak, ViolettaWentz, ElisabetKramer, Eydie N.Wernly, BernhardKranz, SibylleWesolowska, AleksandraKrause, Matthew P.Wessells, RobertKrautwurst, DietmarWessels, IngaKrawinkel, MichaelWesson, DonKrebs, NancyWest, DanielKreider, RichardWestenhoefer, JoachimKrejpcio, ZbigniewWesterterp, MargrietKresty, LauraWesterterp-Plantenga, MargrietKrick, StefanieWewalka, MarleneKristeller, JeanWham, CarolKroese, FloorWhelan, JillKról, EwelinaWhite, John H.Krol, KathleenWhite, Nicole D.Królak-Olejnik, BarbaraWhitfield, JamieKrstic, JelenaWhitfield, KylyKrupa-Kozak, UrszulaWięcek, MagdalenaKrzystek-Korpacka, MalgorzataWięch, PawełKrzyżek, PawełWierzbicka, ElżbietaKsiążek, AnnaWierzejska, ReginaKu, SeockmoWiggs, MichaelKubena, KarenWightman, Emma L.Kucuk, OmerWijesinha-Bettoni, RamaniKuczmarski, MarieWilde, PeteKuhnle, GunterWilk, MichalKujawska, MałgorzataWilkins, HeatherKukreja, SubhashWilkinson, JennyKulke, LouisaWilkowska, AgnieszkaKumar, AmitWillems, MarkKumar, AnandWillette, Auriel A.Kumar, AvinashWilliams, AlisonKumar, BinodWilliams, BarbaraKumar, NitinWilliams, Christopher S.Kumar, PraveenWilliams, Susan LKundu, ParagWilling, BenKundur, AvinashWilmanski, TomaszKung, Woon-ManWilson, CallumKuno, ToshiyaWilson, Jeffrey M.Kunos, GeorgeWilson, PatrickKuo, Ko-LinWilson, TedKupchak, Brian R.Winchester, Laura M.Kupczyński, RobertWinckel, Myriam VanKuppusamy, ManiselvanWinkels, RenateKuroda, KeijiWinpenny, EleanorKurotani, KayoWińska, KatarzynaKurti, StephanieWirnitzer, KatharinaKuryk, LukaszWirth, MichaelKus, PiotrWise, PaulKuwabara, MasanariWiss, DavidKuwabara, TakashigeWitkowska, Anna MariaKwak, Hyun JeongWitkowska-Zimny, MalgorzataKwak, Yi-SubWitkowski, StanisławKwan, Sze Ting (Cecilia)Wittbrodt, MattKwock, Chang KeunWitteman, BenKwok, Sheldon J.J.Włodarczyk, MartaKwok, TimothyWłodarek, DariuszKwon, Eun-YoungWlodek, MaryKwon, HyokjoonWnuk, Susan MargaretKwon, TaehoWoelwer-Rieck, UrsulaKyathanahalli, ChandrashekaraWojdyło, AnetaKyro, CecilieWójkowska, Dagmara WeronikaKyrou, IoannisWölfle, UteKyrozis, AndreasWolfram, EvelynLa Frano, Michael R.Wolnerhanssen, Bettina K.La Manna, GaetanoWolters, MaikeLa Mendola, DiegoWoods-Townsend, KathrynLa Russa, DanieleWoodward, BillLa Vecchia, CarloWoodward, JeremyLa Vieille, SébastienWooten, Joshua S.Laaksonen, OskarWoźniak, LucynaLaatikainen, ReijoWoźniak, ŁukaszLabruna, GiuseppeWright, Charlotte M.Lăcătușu, Cristina-MihaelaWright, HattieLach, GilliardWright, Kenneth P.Lacham-Kaplan, OrlyWrześniok, DorotaLachat, CarlWu, Chang-JiunLachenmeier, DirkWu, Chi-ReiLachlan, MitchellWu, Chung-HsinLachowicz, SabinaWu, JianpingLackner, SonjaWu, Meng-YuLafave, Lynne M.Z.Wu, Ming-JuLaganà, Antonio SimoneWu, RaymondLagoa, RicardoWu, RongxueŁagowska, KarolinaWu, Tzu-HuaLaguzzi, FedericaWu, Wen-TzuLahart, IanWu, ZhihaoLahlil, RachidWulff, Anders BergLahooti, HooshangWullaert, AndyLai, Chih-ChiaWunderlich, Shahla M.Lai, Jun SXaplanteri, PanagiotaLai, YandongXepapadaki, ParaskeviLaird, EamonXia, ChangleiLalor, PatriciaXiang, LanLam, Yan Y.Xiao, HangLam, Yuen TingXiao, XiaLama, AdrianoXiaoli, AlusLamb, MollyXie, WenyanLambert, KellyXu, FurongLamers, YvonneXu, GegeLampiasi, NadiaXu, JiminLamport, Daniel J.Xu, KuiLanari, MarcelloXu, PengfeiLandberg, RikardXu, XiangruLanderos-Weisenberger, AngieXue, HongLandete, José MariaXue, XiangLandry, MatthewYada, ToshihikoLange, EwaYadav, DhananjayLange-Maia, BrittneyYamada, SatoruLangsjoen, PeterYamada, SohsukeLankinen, MariaYAMADA, SonokoLannoy, DamienYamaguchi, MiwaLanza, GiuseppeYamamoto, NorioLanzerstorfer, PeterYamanashi, HirotomoLaparra, Jose MoisésYamashiro, YuichiroLapid, KfirYamashita, AtsushiLappa, IliadaYamazaki, TomomiLara, JoseYan, ShengminLarson Meyer, EnetteYang, Ai-LunLarson, LeilaYang, Chang-HaoLarson, Leila MYang, Cheng-YaoLasekan, John B.Yang, Hsin-YiLassale, CamilleYang, Jae JeongLaster, Scott M.Yang, JianLatino, Maria ElenaYang, JiminLatorre-Moratalla, Mariluz LuzYang, Kuen-ChehLatunde-Dada, Gladys OluyemisiYang, KunLau, Wei LingYang, PeixinLaubach, Victor E.Yang, QiyuanLaughlin, Maren R.Yang, Shi YingLaura Capriotti, AnnaYang, Shwu-HueyLauretani, FulvioYang, Wai YewLaurikka, PilviYang, Wei-hsiungLauschke, Volker M.Yang, XiaoLavelle, FionaYang, YeLavender, AndrewYang, YingLavery, GarethYang, YongkangLaville, MauriceYanni, Amalia E.Lawen, AlfonsYano, ShozoLawley, MeredithYao, HiroshiLawrence-Wood, EllieYasuda, KazukiLe Bon, MelanieYasukazu, TakanezawaLe Dréan, GwenolaYates, Nathanael J.Le Magueresse-Battistoni, BrigitteYau, AdoraLe Nevé, BorisYazbeck, RogerLe Port, AgnesYe, MingyuLê, Kim-AnneYeates, AlisonLea, Michael A.Yeckel, Catherine WeikartLeach, Steven T.Yeh, Kun-yunLeahy, SiobhanYen, Cheng-FangLeal, Ana MendesYen, Chia-HungLeber, BettinaYenerall, JackieLecerf, Jean-michelYeoman, CarlLechner, KatharinaYepuri, Nageshwar R.Leclercq, IsabelleYeruva, LaxmiLedder, OrenYeste, MarcLedeganck, Kristien J.Yeung, Andy Wai KanLederer, Ann-KathrinYeung, EdwinaLederkremer, Gerardo Z.Yi, Joo MiLedo, AnaYin, ChengqianLee, Anne R.Yin, JieLee, Anne RolandYngve, AgnetaLee, BonggiYokomichi, HiroshiLee, Chien-HungYokoyama, HisayoLee, Chun-LinYokoyama, YokoLee, Dong HunYoneshiro, TakeshiLee, ElaineYoo, SeunghyunLee, EunjungYoo, So YoungLee, Hae-JeungYorek, Mark A.Lee, Hong-JinYoshida, HiroshiLee, Hye-JaYoshida, TadashiLee, Hyeon YongYoshikawa, ToruLee, JaecheolYoshikawa, ToshikazuLee, Jae-GilYou, Hyun JuLee, Jen-AiYou, HyunjuLee, Joo HyoungYou, Ren-InLee, Jung EunYoung, AntonyLee, Jun-SooYoung, Richard LLee, KeehoonYoung, WayneLee, KevinYu, Chia-LiLee, KyuwanYu, JiujiuLee, Mi KyungYu, XiaohuaLee, Myoung-SookYu, Xue QinLee, Sang GilYu, Yan PingLee, Seung-YeonYu, YanboLee, SindreYu, YanlinLee, Soo-younYu, YinghuaLee, Sun YoungYuan, LiyunLee, Yen-HanYuan, QiLee, Yi-JangYuan, ZhihongLee, Yoon KwangYui, KunioLee, Yun JungYunus, FakirLee, YunkyoungYusuf, NabihaLeech, RebeccaYutani, ReikoLeemaqz, ShalemYuzyuk, Tatiana N.Leerkes, EstherYzydorczyk, CatherineLefebvre, David E.Zabetakis, IoannisLefere, SanderZabetakis, Ioannis A.Leffler, JonatanZacchia, MiriamLegetic, BrankaZagarins, SofijaLehtisalo, JenniZagórska, AgnieszkaLei, WeiZaguła, GrzegorzLeibovitz, EyalZahradka, PeterLeibowitz, AvshalomZaitsu, MasayoshiLeiherer, AndreasZajac, Ian TaylorLeikin-Frenkel, Alicia I.Zakłos-Szyda, MalgorzataLeimanis, MaraZakrocka, IzabelaLeiphrakpam, PremilaZalewska, MagdalenaLeiva-Brondo, MiguelZalewski, BartlomiejLelli, DianaZambito, YleniaLemay, DanielleZammit, Stefania ChetcutiLemieux, HélèneZamora-Ros, RaulLengyel, ImreZampelas, AntonisLennon, ShannonZang, LiqingLent, Michelle R.Zanini, MilkoLeone, AlessandroZanoli, LucaLeong, Sze YingZanzer, Yoghatama CindyaLeonn Guerrero, RachaelZaprutko, LucjuszLephart, Edwin D.Zaragoza Martí, AnaLerchbaum, ElisabethZárate, RafaelLerin, CarlesZaric, SvetislavLesgards, Jean-FrançoisZarini, GustavoLesicka, MonikaZarobkiewicz, MichałLestavel, SophieZasloff, MichaelLeu, SteveŻebrowska, EwaLevin, SusanZeinstra, GertrudeLevy, LouisZekovic, MilicaLew, Charles Chin HanZeng, HuaweiLewandowska, MałgorzataZeng, MelodyLewis, Erin DianeZeng, NanLewis, MikeZeuner, BirgitteLewis, Nathan AZevallos, Victor F.Lewis, S. J.Zgaga, LinaLi, ChenyuZhang, DeliangLi, Chia-JungZHANG, DUOLi, ChunyingZhang, GuanshiLi, DongmeiZhang, GuodongLi, HuaZhang, PingfengLi, HuinanZhang, QianLi, JieZhang, QiuyangLi, KefengZhang, TuoLi, KeshuaiZhang, WeiLi, LianboZhang, XiaohuiLi, LixinZhang, XuexinLi, MiaomiaoZhang, YixuanLi, Sing-ChungZhang, ZhiyingLi, WeijuanZhao, BaoyinLi, XiaoguangZhao, NingningLi, XinyiZhao, QinLi, YihangZhao, WeiLiang, YanZhao, YanyuLiang, ZhibinZhao, YunLiao, Ling-HsiuZheng, GuopingLiao, Yi-HungZheng, JoleneLiao, Yi-JenZhou, ChiLiao, ZehuanZhou, WenchaoLibra, MassimoZhou, YanjiaoLicari, AmeliaZhu, BingLicciardello, OrazioZhu, HaidongLicciardi, PaulZhu, XiangweiLichtenstein, Mia BeckZhu, YuLiddle, DanyelleZiauddeen, NidaLieder, BarbaraZiegenfuss, TimLieffers, JessicaZiegler, Ekhard ELiepkalns, JustineZiegler, JaneLiévin-Le Moal, VanessaZielińska, DorotaLiew, Chong WeeZielińska, Monika A.Ligat, GaetanZieniuk, BartłomiejLightfoot, AdamZilversmit Pao, LeahLim, Beong OuZimmerman, TahlLim, JunxianZinnai, AngelaLim, KiwonZinöcker, MaritLim, SiewZiobro, RafałLim, Tae-GyuZiouzenkova, OulianaLin, Chih-LiZiser, KatrinLin, Chih-MingZlatan, MujagicLin, Chin-LonZłotkowska, DagmaraLin, Chung-HoZoidis, EvangelosLin, Chung-Tung J.Zordoky, BeshayLin, Hai-ShuZou, Ming-HuiLin, JueZou, PingLin, Muh-ShiZouboulis, Christos C.Lin, Pao-HwaZubarev, RomanLin, Pei-FengZuellig, RichardLin, Pei-YiZukiewicz-Sobczak, WiolettaLin, Sean S-HZuorro, AntonioLin, WenZydowsky, Thomas M

